# Nanovesicles derived from bispecific CAR-T cells targeting the spike protein of SARS-CoV-2 for treating COVID-19

**DOI:** 10.1186/s12951-021-01148-0

**Published:** 2021-11-25

**Authors:** Tianchuan Zhu, Yuchen Xiao, Xiaojun Meng, Lantian Tang, Bin Li, Zhaoyan Zhao, Qingqin Tan, Hong Shan, Lei Liu, Xi Huang

**Affiliations:** 1 Center for Infection and Immunity, The Fifth Affiliated Hospital of Sun Yat-Sen University, Zhuhai, 519000 Guangdong China; 2 Guangdong Provincial Key Laboratory of Biomedical Imaging, The Fifth Affiliated Hospital of Sun Yat-Sen University, Zhuhai, 519000 Guangdong China; 3 Southern Marine Science and Engineering Guangdong Laboratory, Zhuhai, 519000 Guangdong China; 4 Shenzhen Key Laboratory of Pathogen and Immunity, National Clinical Research Center for Infectious Disease, Shenzhen Third People’s Hospital, Shenzhen, 518112 Guangdong China

**Keywords:** COVID-19, nanovesicles, Neutralizing antibody, Remdesivir, Targeted delivery

## Abstract

**Background:**

Considering the threat of the COVID-19 pandemic, caused by SARS-CoV-2, there is an urgent need to develop effective treatments. At present, neutralizing antibodies and small-molecule drugs such as remdesivir, the most promising compound to treat this infection, have attracted considerable attention. However, some potential problems need to be concerned including viral resistance to antibody-mediated neutralization caused by selective pressure from a single antibody treatment, the unexpected antibody-dependent enhancement (ADE) effect, and the toxic effect of small-molecule drugs.

**Results:**

Here, we constructed a type of programmed nanovesicle (NV) derived from bispecific CAR-T cells that express two single-chain fragment variables (scFv), named CR3022 and B38, to target SARS-CoV-2. Nanovesicles that express both CR3022 and B38 (CR3022/B38 NVs) have a stronger ability to neutralize Spike-pseudovirus infectivity than nanovesicles that express either CR3022 or B38 alone. Notably, the co-expression of CR3022 and B38, which target different epitopes of spike protein, could reduce the incidence of viral resistance. Moreover, the lack of Fc fragments on the surface of CR3022/B38 NVs could prevent ADE effects. Furthermore, the specific binding ability to SARS-CoV-2 spike protein and the drug loading capacity of CR3022/B38 NVs can facilitate targeted delivery of remdesiver to 293 T cells overexpressing spike protein. These results suggest that CR3022/B38 NVs have the potential ability to target antiviral drugs to the main site of viral infection, thereby enhancing the antiviral ability by inhibiting intracellular viral replication and reducing adverse drug reactions.

**Conclusions:**

In summary, we demonstrate that nanovesicles derived from CAR-T cells targeting the spike protein of SARS-COV-2 have the ability to neutralize Spike-pseudotyped virus and target antiviral drugs. This novel therapeutic approach may help to solve the dilemma faced by neutralizing antibodies and small-molecule drugs in the treatment of COVID-19.

**Graphical Abstract:**

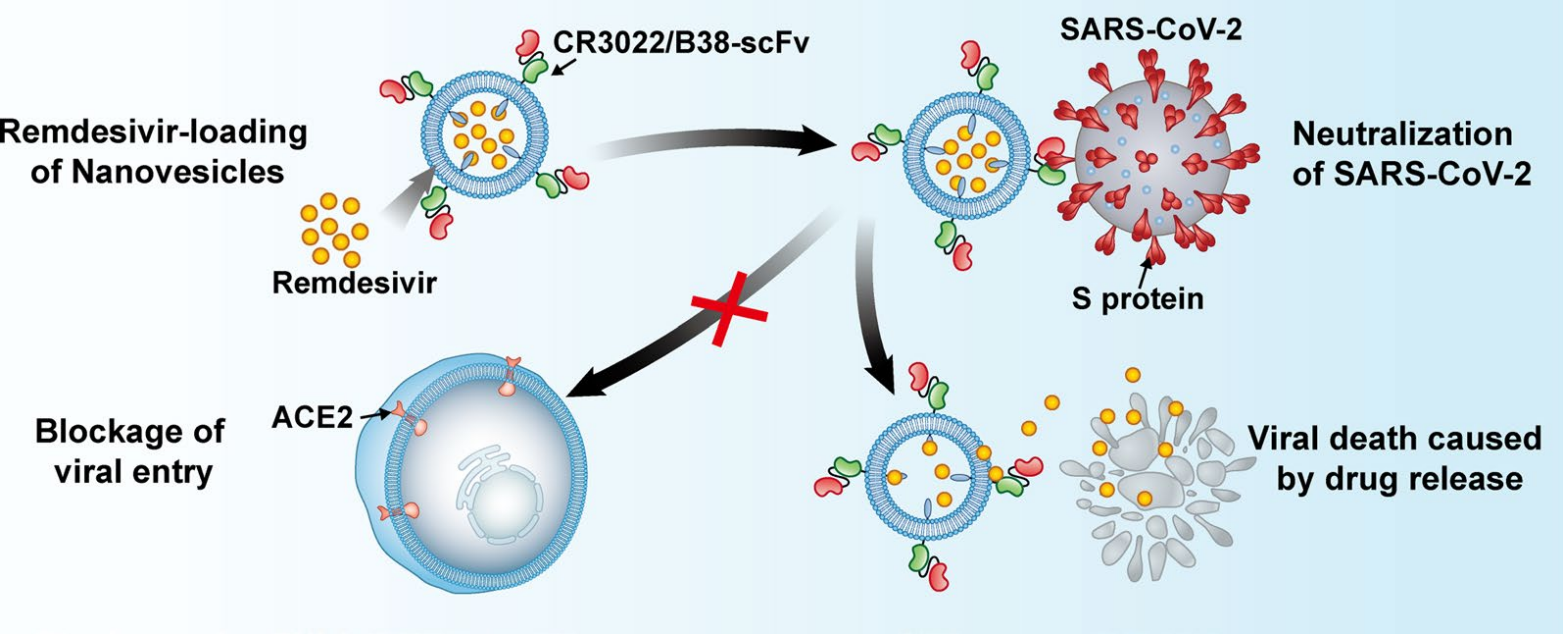

**Supplementary Information:**

The online version contains supplementary material available at 10.1186/s12951-021-01148-0.

## Introduction

As of October 1, 2021, over 234 million cases of coronavirus disease 2019 (COVID-19) have been confirmed, and over 4.7 million deaths have been reported globally (https://www.worldometers.info/coronavirus/). It is necessary to develop an effective vaccine and treatment to solve this global public health challenge.

Currently, neutralizing antibodies and remdesivir are considered promising drugs for the treatment of COVID-19, and many studies have evaluated their safety and effectiveness [1–3]. Previous studies have confirmed that both the neutralizing antibody CR3022 and B38 neutralize SARS-CoV-2 infection. CR3022 and B38 were isolated from a convalescent SARS-CoV-1 patient and a SARS-CoV-2 patient, respectively [4, 5]. However, under the selective pressure of a single neutralizing antibody, the SARS-CoV-2 strain may resist the neutralizing effect through the accumulation of spontaneous mutations [6]. Mutations have been detected within the epitopes for the neutralizing antibodies S309 (N354D/K and S359N) and VHH-72 (R408I, K378R, and P384L) [6]. Although the frequency of these mutations is low, continuous treatment with a single neutralizing antibody may render any of these mutated SARS-CoV-2 strains dominant. In addition, the viral-antibody complexes can be endocytosed by macrophages through the binding of the Fc fragment to Fc receptors, thereby causing a severe antibody-dependent enhancement (ADE) effect that can promote viral infection [6, 7]. Considering that the ADE effect has been shown at the cellular level mediated by SARS-CoV-2-specific antibodies [8–10], the potential for an ADE effect should be considered when using neutralizing antibodies to treat COVID-19. Furthermore, the recommended dose of intravenous remdesivir has limited benefit in COVID-19 patients due to the low drug concentration in the lungs, while excessive doses (≥ 200 mg per day) may cause systemic adverse reactions in COVID-19 patients such as liver toxicity [11, 12]. Therefore, a new treatment approach for COVID-19 is needed to overcome these problems.

Vesicles derived from cell membranes, such as exosomes, macrovesicles, and vesicles extruding from the cell membrane, play an important role in the targeted delivery of biotherapeutics due to their biocompatibility, modification, and ability to cross biological barriers [13, 14]. One common method of enhancing their targeting ability is to express ligands or chimeric antigen receptors (CARs) on the surfaces of vesicles that specifically bind to the target cells [15]. As previously reported, engineered nanovesicles derived from 293 T cells expressing PD-1 can target loaded 1-methyl-tryptophan to melanoma cells that express large quantities PD-L1, to achieve synergistic killing of tumor cells [16]. Exosomes derived from EGFR CAR-T cells also showed good targeting properties for solid tumors with EGFR overexpression [17]. In addition, it has recently been reported that both Spike CAR-macrophage cells and Spike CAR-NK cells have shown good targeting and neutralizing ability to SARS-CoV-2 pseudotyped virus [18, 19]. However, considering that cytokine release syndrome (CRS) may occur when CAR-immune cell therapy is used for viral infections, it may be a safer choice to prepare CAR-immune cells into nanovesicles. Nanovesicles can not only inherit the ability of cells expressing CAR to target SARS-CoV-2, but also have the ability to load antiviral drugs.

**Scheme 1.**
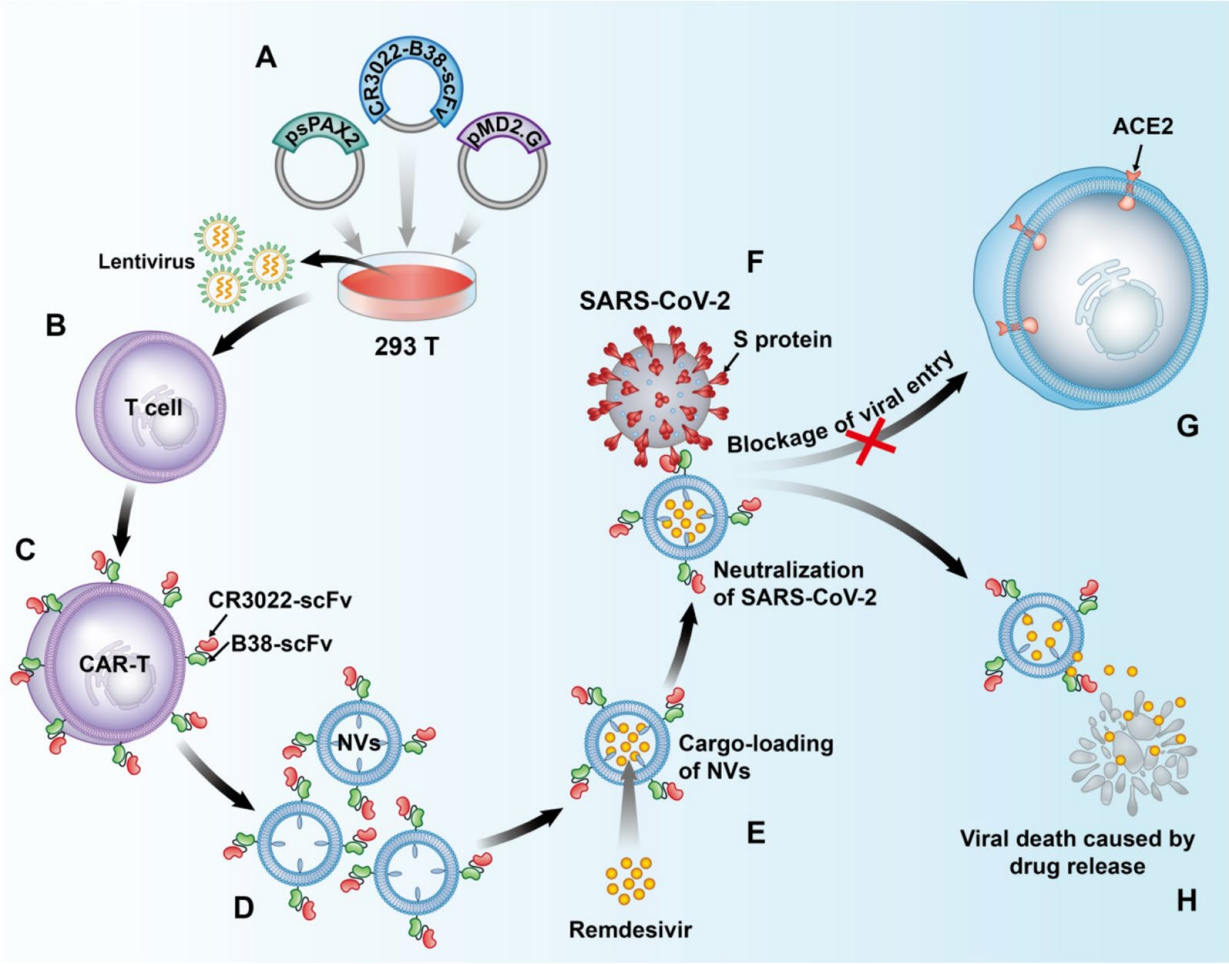
Schematic illustration of nanovesicles derived from bispecific CAR-T cells targeting the spike protein of SARS-CoV-2 for treating COVID-19. **A**, **B** Lentiviruses encoding CR3022/B38 CAR were obtained using a triplasmid lentivirus packaging system. **C** Lentiviruses infected T cells and CR3022/B38 CAR-T cells were collected. **D** Nanovesicles expressing CR3022/B38 scFv were prepared by extrusion. **E** CR3022/B38 NVs loaded with remdesivir by electroporation. **F**–**G** CR3022/B38 NVs prevented SARS-CoV-2 from entering cells expressing ACE2 at high levels by recognizing and binding specifically to the spike protein of SARS-CoV-2. **H** Remdesivir was released from CR3022/B38 NVs in the major sites of viral infection, and synergistically eliminated SARS-CoV-2 by inhibiting intracellular virus replication

Inspired by targeted delivery of anti-cancer drugs and the antiviral application of immune cells, we designed a new type of programmed nanovesicle derived from bispecific CAR-T cells. Here, the T cell is used as a model cell to prepare nanovesicles with the ability to neutralize viruses and carry drugs. These nanovesicles express two single-chain fragment variables (scFv), named CR3022 and B38 (Scheme 1). CR3022 recognizes the conserved epitopes of the S protein of SARS-CoV-1 and SARS-CoV-2, and B38 recognizes the RBD region of SARS-CoV-2 and blocks the binding between the virus and the ACE2 receptor [4, 20]. The use of antibody cocktail therapy during viral infection can significantly reduce the incidence of resistance [21, 22]. Hence, the co-expression of CR3022 and B38 targeting different epitopes of S protein, not only enhances the neutralizing ability of CR3022/B38 NVs, but also enables CR3022/B38 NVs to retain the ability to neutralize SARS-CoV-2 even after mutations occur in certain S protein epitopes. Moreover, the lack of Fc fragments on the nanovesicles could prevent the ADE effect mediated by macrophage endocytosis, thus preventing cytokine storms. Remdesivir was encapsulated in CR3022/B38 NVs by electroporation and then delivered into the infectious sites of SARS-CoV-2 based on CR3022/B38 NVs targeting. In this manner, CR3022/B38 NVs loaded with remdesivir have the ability to neutralize extracellular viruses and inhibit intracellular virus replication through the released remdesivir. Targeted delivery allows remdesivir to reach a higher drug concentration in the infectious sites such as the lung at the recommended dosage, so as to achieve the goal of synergistically eliminating SARS-CoV-2 and reducing systemic side effects.

## Results and discussion

### Preparation and characterization of CAR-T cells for nanovesicles

**Fig. 1:**
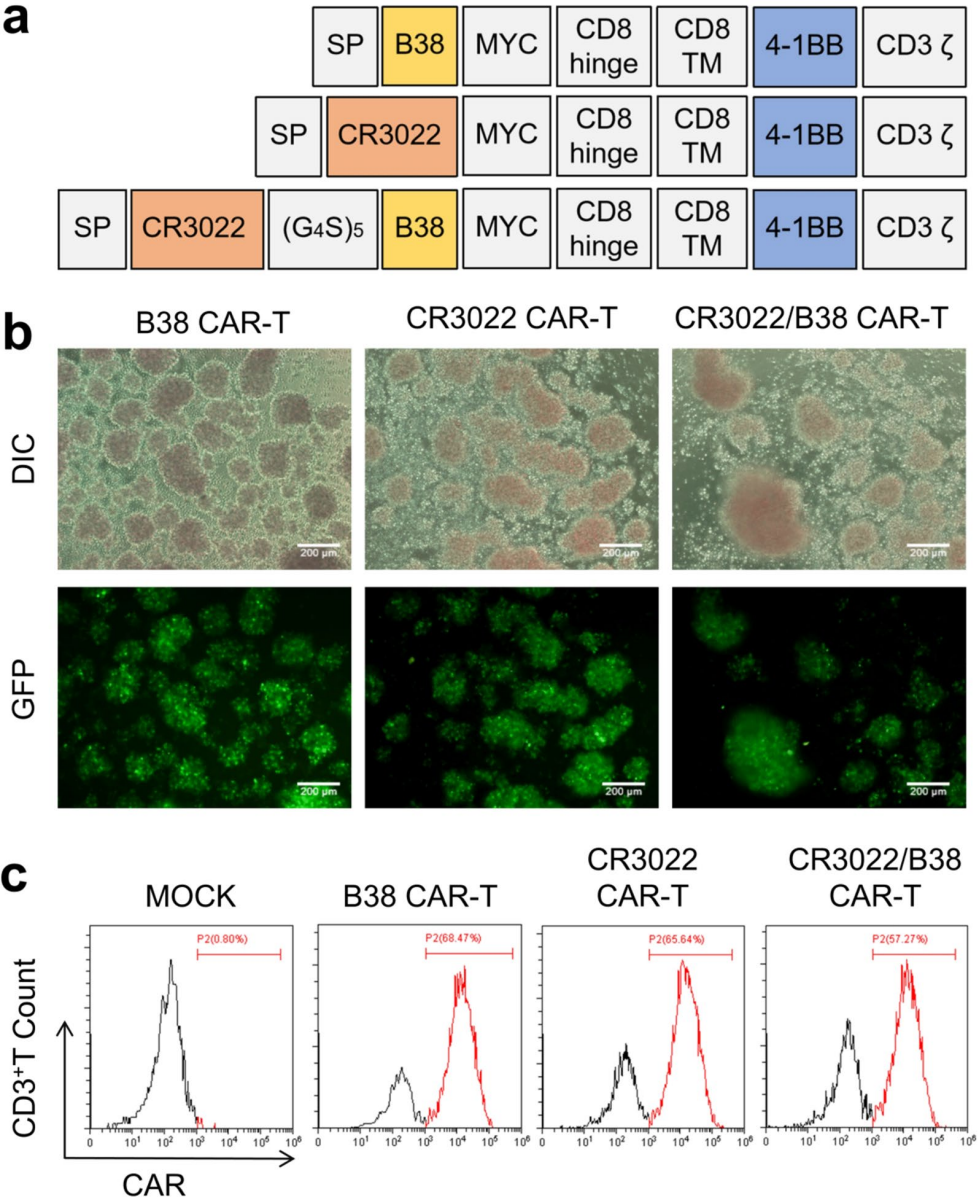
Preparation and characterization of three types of CAR-T cells. **a** Molecular design of CR3022 CAR, B38 CAR, and CR3022/B38 CAR. MYC was used as a protein label for Western blotting. **b** Lentivirus transfection efficiency in T cells was observed by fluorescence microscopy. **c** Representative flow cytometry histogram. CAR expression in T cells was detected using protein L

To investigate our hypothesis, we designed CAR-T cells expressing CR3022, B38, and CR3022/B38 (Fig. 1a). CR3022 and B38 were synthesized by BGI according to the published sequences. The following MYC protein tag is widely used in Western blotting as a protein label. Additionally, the intracellular domain is based on the classical CD19 CAR-T design [23]. We subcloned the sequences encoding these CARs into a lentiviral backbone plasmid and then co-transfected them with helper plasmids into 293 T cells to produce CAR lentiviruses. As a result, CAR-T cells expressing scFv were collected by infecting T cells isolated from peripheral blood with lentiviruses encoding the CARs. Because the CAR lentivirus also contains a gene encoding green fluorescent protein (GFP), the lentivirus' transduction efficiency in T cells can be indirectly observed under an inverted fluorescence microscope. The expected high transduction efficiency occurred in three CAR lentiviruses in T cells (Fig. 1b). The fluorescence of CR3022/B38 CAR-T was slightly lower than that of the other counterparts, possibly due to the longer gene sequence encoding CR3022/B38 CAR, which affects the expression of the downstream GFP gene. Furthermore, scFv expression was detected by flow cytometry, and the transduction efficiency of the lentivirus was estimated to be between 57.27 and 68.47% (Fig. 1c), similar to the percentage determined using the inverted fluorescence microscope. These results indicated that scFv targeting the S protein was successfully expressed in T cells.

### Preparation and characterization of nanovesicles

**Fig. 2:**
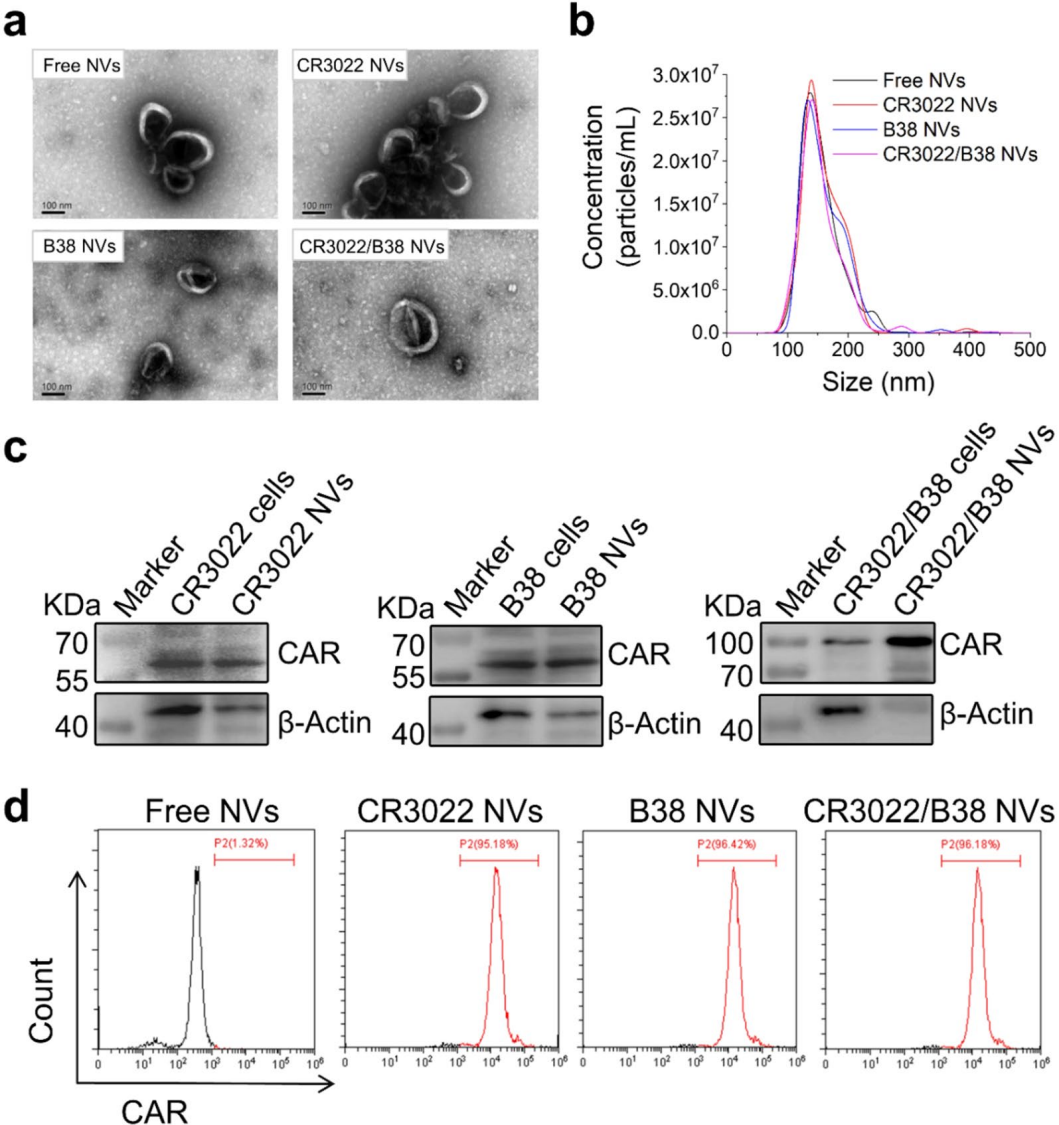
Preparation and characterization of nanovesicles derived from three types of CAR-T cells. **a** The morphology and particle size of the three types of nanovesicles obtained by TEM. Scale bar: 100 nm. **b** Determination of the size distribution of the three types of nanovesicles through NTA. **c** The expression of CAR protein in nanovesicles and whole-cell lysates of the three types of CAR-T cells analyzed by Western blotting. **d** Flow cytometry showing the expression of scFv on the nanovesicles conjugated to latex beads

Next, we separated the cell membrane from the three mentioned CAR-T cells by lysis and physical grinding [16, 24], followed by dispersing the isolated membranes in phosphate buffered saline (PBS; pH 7.4). After ultrasonic processing, the nanovesicles were obtained by serial extrusion (20 rounds) through 800 nm and 200 nm polycarbonate membranes using an extruder. The yield of nanovesicles was approximately 19.5 μg of total protein and 27.3 × 10^9^ particles per 1 × 10^6^ CAR-T cells, which was more than 100-fold natural secreted exosomes. Transmission electron microscopy (TEM) and nanoparticle tracking analysis (NTA) (Fig. 2a, b) showed that the nanovesicles had good homogeneity, and that the particle diameter peaked at approximately 140 nm. To further indicate the expression of scFv targeting the S protein in the purified nanovesicles, Western blotting was performed on the three types of nanovesicles and whole-cell lysates of the related CAR-T cells. The results were as expected (Fig. 2c). In addition, to ensure that the scFv on the nanovesicles could successfully bind to the S protein, the antigen-binding domain of scFv on the nanovesicles must always be outwards. Therefore, we conjugated the nanovesicles to latex beads and used flow cytometry to prove that the scFv was outwards on the nanovesicles (Fig. 2d).

### Targeting of nanovesicles to the spike protein of SARS-CoV-2

**Fig. 3:**
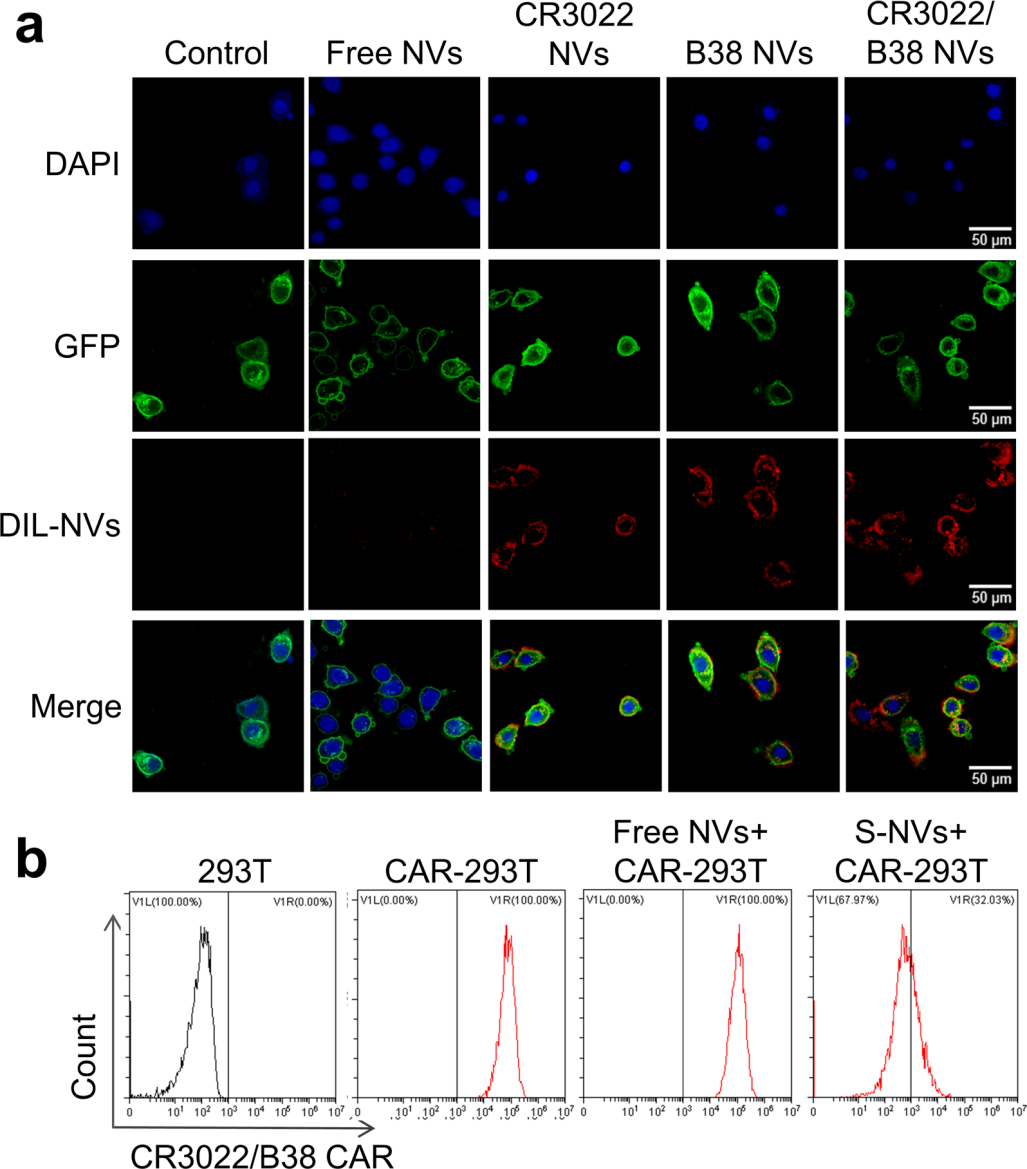
Specific binding capacity of the three types of nanovesicles to the spike protein in vitro. **a** Dil labeled nanovesicles (red) and 293 T-S cells (green) were co-incubated in vitro for 4 h, and the localization of the nanovesicles and S protein was observed by confocal microscopy. CR3022 NVs, B38 NVs, and CR3022/B38 NVs showed a higher binding affinity to the S protein than free NVs. Scale bar: 50 nm. **b** S-NVs were co-incubated with CR3022/B38 CAR-293 T cells for 2 h followed by flow cytometry analysis. 293 T cells, CAR-293 T cells and free NVs + CAR-293 T cells were set as controls

The scFv on nanovesicles could neutralize the virus by recognizing and binding to the S protein of SARS-CoV-2. To explore the targeting ability of the three types of nanovesicles to S protein, we co-incubated the three types of nanovesicles with 293 T cells expressing S protein (293 T-S cells) in vitro. We obtained 293 T-S cells under puromycin selection pressure after infection with lentivirus containing the S protein gene sequence. The lentivirus also harbored a gene sequence encoding a GFP protein anchored on the cell membrane, which reflects a green fluorescent signal to the cell membrane of 293 T-S cells (Additional file 1: Fig. S1a). The presence of S protein was confirmed by Western blot analysis (Additional file 1: Fig. S2b). In addition, Dil dye and DAPI were used to provide a red fluorescent label for nanovesicles and a purple fluorescent label for the nuclei of 293 T-S cells, respectively. Confocal microscopy showed (Fig. 3a) that after 4 h of co-incubation, all three types of nanovesicles could effectively bind to the surface of 293 T-S cells, while the binding efficiency was much lower for nanovesicles not expressing scFv targeting S protein (free NVs).

A blocking experiment was performed for nanovesicles expressing S protein (S-NVs) to confirm that the binding was antigen-specific (Fig. 3b). To make the blocking experimental results more stable, we used CR3022/B38 CAR lentiviruses containing puromycin genes to prepare CR3022/B38 CAR-293 T cells that can more stably express scFv to replace CAR-T cells. CR3022/B38 CAR-293 T cells were co-incubated with S-NVs for 2 h, to determine whether the antigen-binding domain of scFv was blocked by S-NVs. Flow cytometry showed that S-NVs could block the antigen-binding domains on CR3022/B38 CAR-293 T cells better than free NVs. These results indicated that these three types of nanovesicles could recognize and bind S protein, and that binding was antigen-specific.

### Nanovesicle neutralization assay

**Fig. 4:**
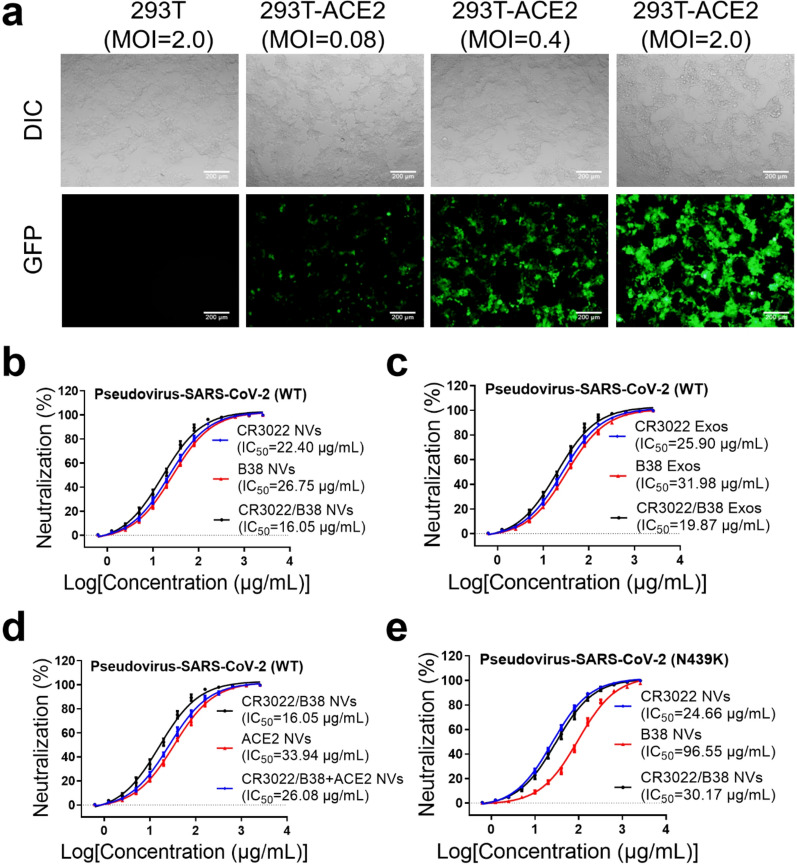
Three types of nanovesicles effectively neutralized the spike-pseudotyped virus. **a** 293 T and 293 T-ACE2 cells infected with the packaged spike-pseudotyped virus at different MOIs. Scale bar: 200 nm. **b** Serial twofold dilution of the three types of nanovesicles were co-incubated with the spike pseudoviruses (wild type, WT) for 10 min, and then the mixture was added to 293 T-ACE2 cells. After 36 h, the IC50 of the three types of nanovesicles was detected by measuring the luciferase expression levels in 293 T-ACE2 cells. **c** The IC50 of CR3022 Exos, B38 Exos and CR3022/B38 Exos was detected by measuring the luciferase expression levels in 293 T-ACE2 cells. **d** CR3022/B38 and ACE2 NVs are used in combination to neutralize the spike pseudoviruses (WT). **e** The IC50 of neutralization of the spike pseudoviruses (N439K mutant) by CR3022 NVs, B38 NVs, and CR3022/B38 NVs

Because SARS-CoV-2 must be cultured in a Biosafety Level 3 laboratory, we used pseudotyped lentiviral particles with SARS-CoV-2 spike protein to test the neutralization of the three types of nanovesicles [25]. Spike-pseudotyped virus was generated through the HIV lentivirus system, in which the nucleotide sequence of the spike protein was taken from the SARS-CoV-2 strain Wuhan-hu-1 (Genbank NC_045512). The expression plasmid of the lentivirus system contained both luciferase and GFP genes to allow researchers to observe the process by which the pseudovirus enters cells. Additionally, 293 T cells with high expression of ACE2 (293 T-ACE2 cells) were prepared by lentiviral vectors encoding ACE2, which were treated as target cells for pseudovirus infection. The presence of ACE2 protein was confirmed by western blotting (Additional file 1: Fig. S1c). The results of the inverted fluorescence microscope showed that the spike-pseudotyped virus can only infect 293 T-ACE2 cells, indicating that the packaging of the pseudovirus had been successful (Fig. 4a).

Serial twofold dilutions of the three types of nanovesicles were incubated with pseudovirus for 10 min, respectively. The mixture was then added to 293 T-ACE2 cells at 2 × 10^4^ cells per well for 36 h. The quantitative detection of luciferase showed that the half maximal inhibitory concentration (IC50) of the three types of nanovesicles to neutralize the pseudovirus was between 16.05 and 26.75 μg/mL (Fig. 4b). Notably, the neutralization ability of CR3022/B38 NVs was slightly stronger than that of the other two nanovesicles, indicating that the antibodies that recognize different epitopes of S protein likely have a superposition effect. Mechanistically, the enhanced neutralization of bi-specific nanovesicles may be attributed to the improved avidity of CAR protein on the surface [26, 27]. Affinity describes the binding strength of a single bond, whereas avidity equals the total binding strength of a CAR protein at each binding site. Thus, though the affinity of CR3022 and B38 scFvs is unchanged, the bi-specific CAR protein containing these two scFvs could possess stronger avidity as they simultaneously bind multiple epitopes of RBD protein.

In addition, the results of the neutralization experiment show that the three types of CAR-T cell-derived exosomes also have a good ability to neutralize the spike pseudotyped viruses (Fig. 4c). As CAR-T cell-derived exosomes can inhibit virus replication through granzymes [17], their ability to eliminate viruses may be stronger than that of nanovesicles. Furthermore, previous reports confirmed that recombinant ACE2 protein and ACE2 NVs could block SARS-CoV-2 from entering cells that express high levels of ACE2 [28–30]; thus, we used 293 T-ACE2 cells to prepare ACE2-NVs for virus neutralization experiments. The results show that the neutralizing ability of ACE2-NVs was worse than that of CR3022/B38 NVs, and that the neutralizing ability of the two nanovesicles was not significantly improved after the combination (Fig. 4d). However, it should be noted that since ACE2 has different affinities for the S proteins of different SARS-CoV-2 variants, the neutralizing ability of ACE2 NVs to different variants may also vary [31, 32]. Furthermore, considering that ACE2 and the scFvs against S protein have independent mechanisms to recognize the S protein, the co-expression of ACE2 and scFvs on nanovesicles may be beneficial when SARS-CoV-2 is mutated. Moreover, we used the three types of CAR-293 T cells to prepare CR3022, B38, and CR3022/B38-293 T NVs for virus neutralization experiments. Although they may not be as good as nanovesicles derived from T cells in terms of safety and patient acceptance, CR3022/B38-293 T NVs showed a stronger ability to neutralize spike-pseudotyped virus than CR3022/B38 CAR-T NVs (Additional file 1: Fig. S2). Finally, we tested the ability of the three types of nanovesicles to neutralize pseudotyped N439K mutant virus. Consistent with a previous report [33], the N439K mutant significantly decreased the neutralization sensitivity of the virus to B38 NVs, but retained sensitivity to CR3022/B38 NVs (Fig. 4e). These results indicated that nanovesicles expressing bispecific monoclonal antibodies may help to prevent the escape of virus strains with a single mutation.

### Nanovesicles target remdesivir to the spike protein of SARS-CoV-2

**Fig. 5:**
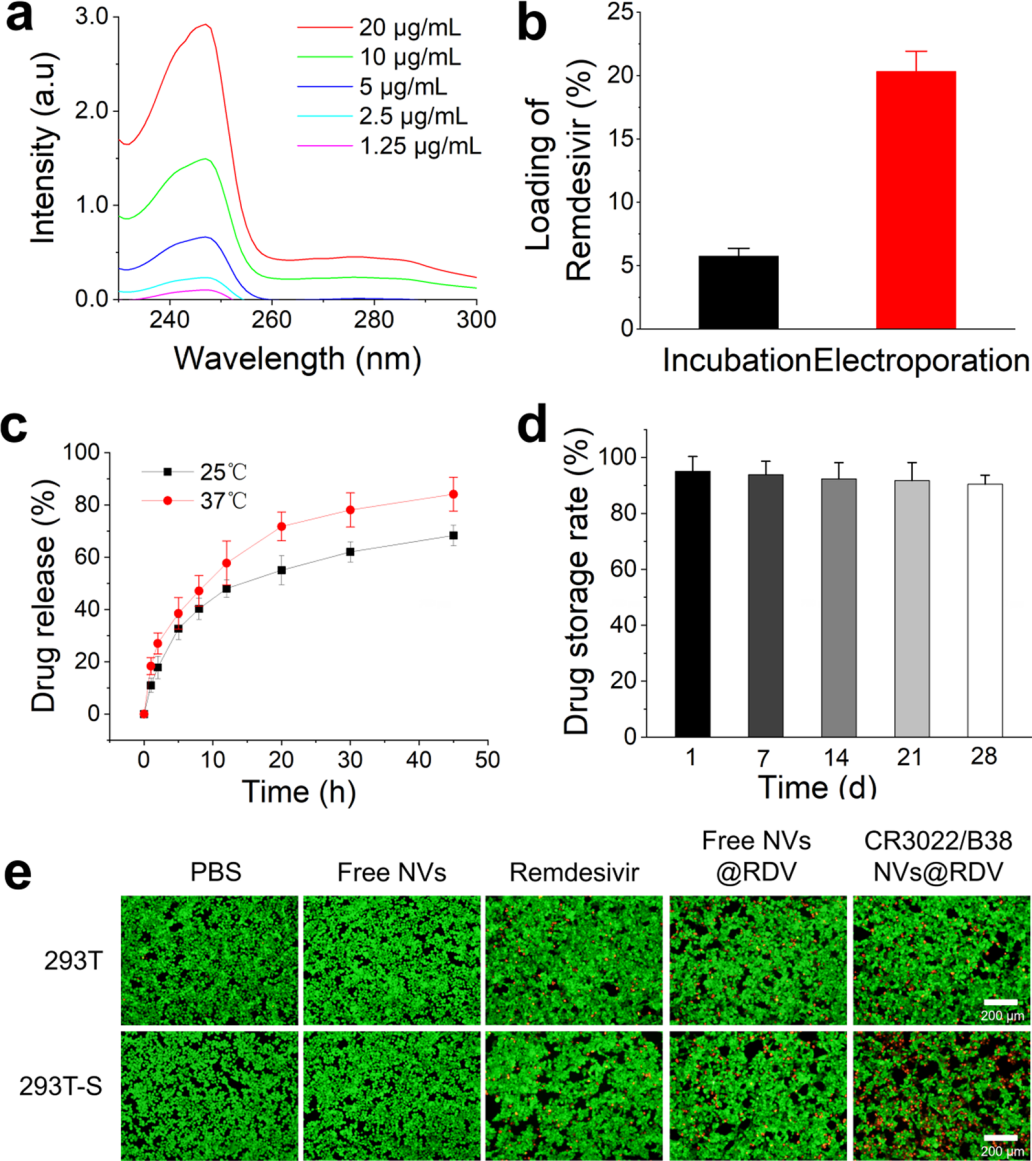
Delivery efficiency of remdesivir by CR3022/B38 NVs to 293 T-S cells. **a** Determination of the standard curve for detecting remdesivir using a spectrophotometer. Mass of remdesivir (μg/mL) = 6.5507 OD value of test sample + 0.6505. **b** Packaging efficiency of remdesivir loading into nanovesicles using different methods. Approximately 2 μg of remdesivir was encapsulated in 10 μg of nanovesicles using electroporation. **c** Drug release profile of remdesivir-loaded nanovesicles at different temperatures. The percentage of drug release (%) = OD value of the remdesivir released from the nanovesicles/OD value of the total remdesivir in the NVs × 100%. **d** Remdesivir remaining in the thawed nanovesicles. The nanovesicles were frozen at − 80 °C, before thawing at different time points, and measuring the remdesivir remaining in the nanovesicles. **e** PBS, free NVs, remdesivir, remdesivir-free NVs, and remdesivir-CR3022/B38 NVs were co-incubated with 293 T or 293T-S cells, respectively, followed by Calcein-AM/PI staining and detection by fluorescence microscopy. Scale bar: 200 nm

After studying the targeting and neutralization ability of the three types of nanovesicles, we loaded remdesivir into CR3022/B38 NVs by electroporation to determine whether they can perform targeted delivery of antiviral drugs. The quantization of remdesivir was determined by spectrophotometry, showing that remdesivir had a specific absorption peak at 247 nm. When the concentration of remdesivir was between 1.25 and 20 μg/mL, the trend still conformed to the standard curve (Fig. 5a). The drug loading rate of remdesivir in CR3022/B38 NVs (mass ratio of remdesivir/NVs protein) was also determined by a spectrophotometry. Spectrophotometry showed that electroporation was associated with a higher drug loading rate (≈ 20.3%) of remdesivir in nanovesicles than simple co-incubation of remdesivir nanovesicles were (≈ 5.8%) (Fig. 5b). After loading of remdesivir, the zeta potential of nanovesicles decreased from − 24.96 mV to − 27.56 mV (Additional file 1: Fig. S3). In addition, drug release experiments showed that remdesivir in nanovesicles could release more than half of the stored amount within 10 h at 37 °C (Fig. 5c). In order to account for drug leakage from nanovesicles, we assessed the stability of CR3022/B38 NVs loaded with remdesivir stored at − 80 °C. Stability studies indicated that the amount of remdesivir remaining in the thawed nanovesicles after 28 days of storage was > 90% (Fig. 5d). The particle size of the nanovesicles did not change significantly after 28 days of storage at − 80 °C (Additional file 1: Fig. S4). Moreover, the nanovesicles showed good stability in PBS containing 20% fetal bovine serum (FBS) (Additional file 1: Fig. S5), indicating that these nanovesicles may be good carriers for drug delivery.

Because the drug delivery of CR3022/B38 NVs was achieved by targeting the SARS-CoV-2 S protein, we constructed 293 T-S cells to replace SARS-CoV-2. Due to the cytotoxicity of remdesivir, if CR3022/B38 NVs can deliver more remdesivir to 293 T-S cells within a certain time than other experimental groups, it will induce more 293 T-S cells death. After co-incubation with different nanovesicles or remdesivir, the survival rate of target cells was determined using Calcein-AM/PI staining, in which green light represented living cells and dead cells appeared yellow. Staining showed that CR3022/B38 NVs loaded with remdesivir caused more death of 293 T-S cells (Fig. 5e). In addition, the survival rate of target cells in different treatment groups was quantified by ImageJ software. Quantitative results showed that the survival rate of 293 T-S cells incubated with CR3022/B38 NVs loaded with remdesivir was approximately 71.07% (Additional file 1: Fig. S6), which was much lower than that of other groups. These results demonstrated that CR3022/B38 NVs have well-targeted delivering capability.

### Targeting and neutralization of nanovesicles in vivo

**Fig. 6:**
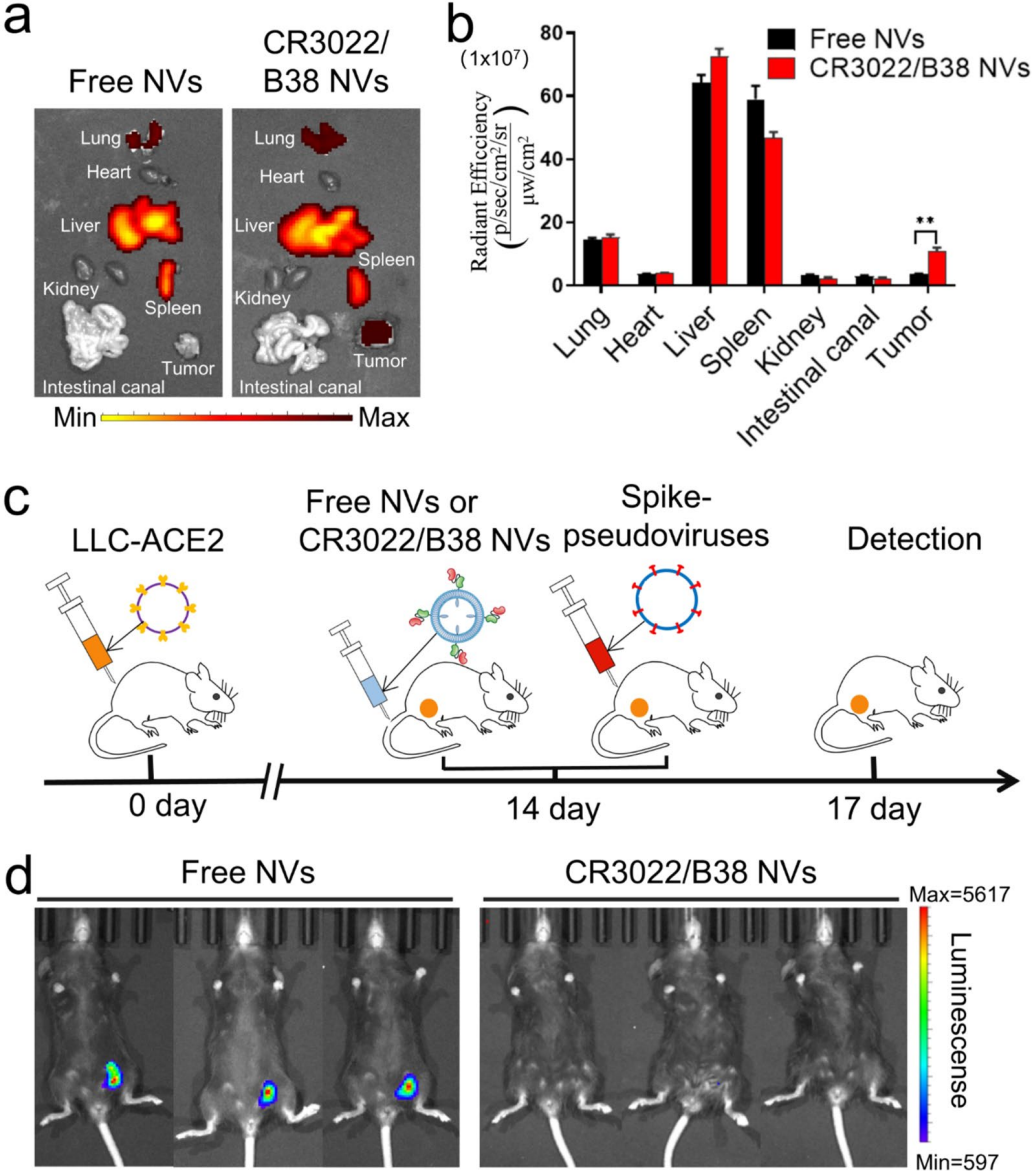
Targeting and neutralization ability of CR3022/B38 NVs in vivo. **a**, **b** Free NVs and CR3022/B38 NVs labeled with DiR were injected into LLC-S tumor-bearing mice through the tail vein, and the accumulation of free NVs and CR3022/B38 NVs in mouse organs and tumors was detected at 12 h post-injection by IVIS system (*n* = 3, error bar, mean ± s.d). **P < 0.01. **c** Flow chart for the spike-pseudotyped virus-based neutralization assay in vivo. Free and CR3022/B38 NVs were injected into LLC-ACE2 tumor-bearing mice via the tail vein, and Spike-pseudotyped viruses were injected intratumorally 2 h later. The amount of pseudoviruses entering LLC-ACE2 tumors 72 h post-injection was determined by fluorescence intensity. **d** IVIS imaging of mice after spike-pseudotyped virus-based neutralization assay depicted in (**c**)

To explore whether CR3022/B38 NVs also have good targeting and viral neutralization ability in vivo, Lewis lung carcinoma (LLC) cells with stable and highly expressed S protein (LLC-S cells) or ACE2 protein (LLC-ACE2 cells) were prepared and planted in the groins of C57 mice. After 14 days, DiR-labeled CR3022/B38 NVs were injected into LLC-S tumor-bearing mice through the tail vein, and the tissue distribution of CR3022/B38 NVs in the mice was measured at 12 h post-injection. It was observed that CR3022/B38 NVs accumulated mainly in the liver, spleen, lung, and tumor sites (Fig. 6a, b). In contrast, there was almost no accumulation of free NVs in tumor sites, indicating that CR3022/B38 NVs retained significant targeting ability in a complex environment in vivo.

To investigate the ability of CR3022/B38 NVs to neutralize SARS-CoV-2 in vivo, we constructed a SARS-CoV-2 pseudovirus/mouse challenge model. The pseudovirus/mouse challenge model has been widely used to evaluate the effectiveness of vaccines or neutralizing antibodies to protect the body from virus invasion [34–36]. To test the neutralization of CR3022/B38 NVs in vivo, the spike-pseudotyped viruses encoding luciferase were injected into LLC-ACE2 tumors, followed by CR3022/B38 NV administration at different time points. At 72 h post-injection of the spike-pseudotyped virus, the expression of luciferase in LLC-ACE2 tumor tissue was measured (Fig. 6c). Notably, no fluorescence was observed in the mice injected with CR3022/B38 NVs, suggesting that CR3022/B38 NVs neutralized most of the spike-pseudotyped viruses (Fig. 6d). In contrast, free NVs failed to block the spike-pseudotyped virus from entering ACE2-LLC cells and thus induced strong fluorescent expression in ACE2-LLC tumor tissue. Besides, even when CR3022/B38 NV injection was given 12 h in advance, spike-pseudotyped virus invasion was still blocked, indicating the long half-life of NVs in vivo (Additional file 1: Fig. S7).

### Safety evaluation of nanovesicles

**Fig. 7:**
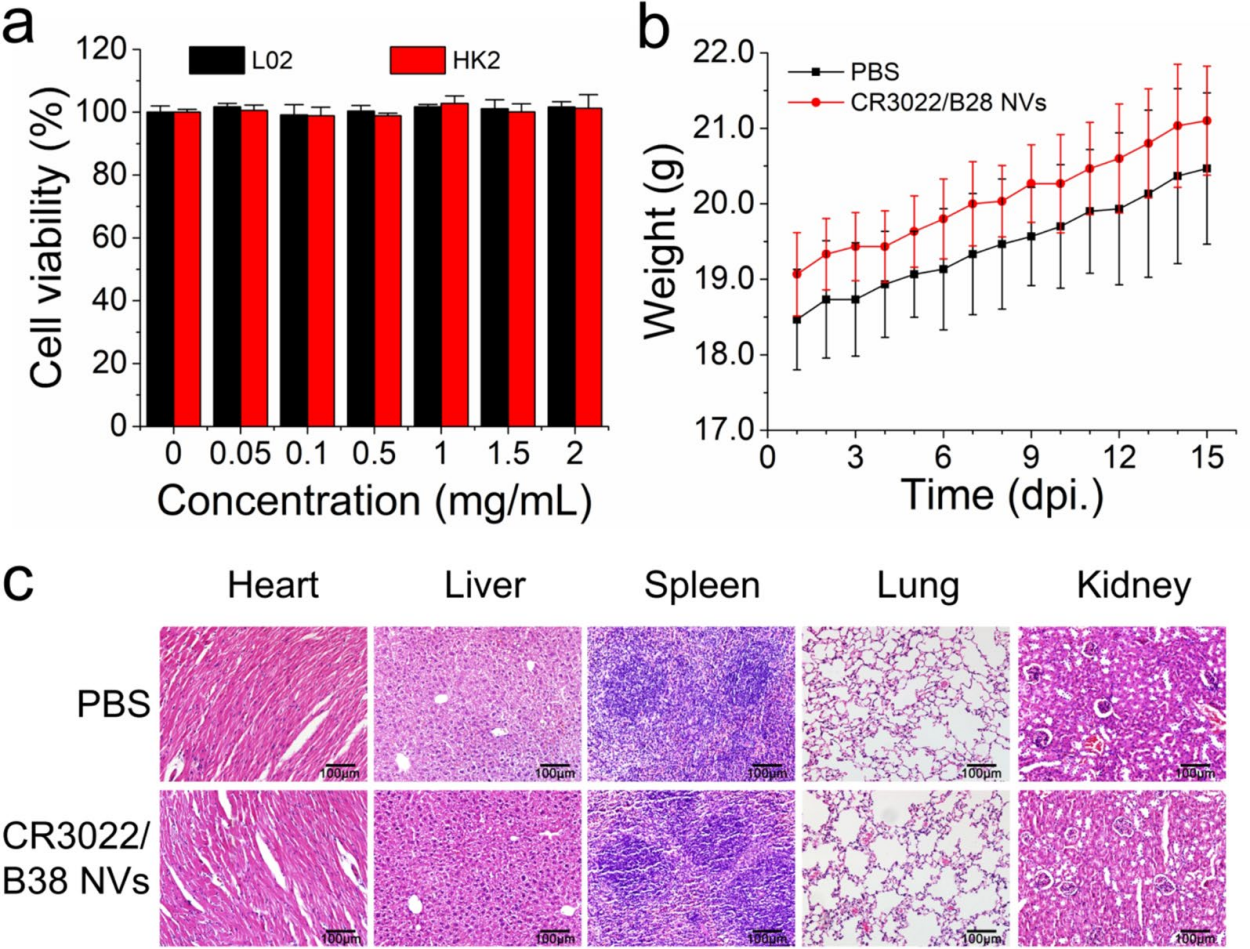
Safety evaluation of CR3022/B38 NVs. **a** The cell viability of L02 cells or HK-2 cells co-cultured with CR3022/B38 NVs was measured by CCK-8 assay. **b** Body weight curves of the mice treated with CR3022/B38 NVs or PBS. **c** H&E stained histological sections of the heart, liver, spleen, lung, and kidney from mice treated with CR3022/B38 NVs or PBS. Scale bar: 100 μm

To further evaluate the safety of CR3022/B38 NVs, we performed several assays, including cytotoxicity assays, and examined the changes in body weight and histopathology of the mice treated with CR3022/B38 NVs. The results of the CCK-8 assay showed that, in the CR3022/B38 NVs concentration range of 0.05–2 mg/mL, the cell viability was higher than 99% in the human normal liver (L02) cells and human proximal tubular epithelial (HK-2) cells (Fig. 7a). The body weight of the mice treated with CR3022/B38 NVs exhibited an upward trend (Fig. 7b), which was not significantly different from the control group. Moreover, after CR3022/B38 NV treatment in vivo, no pathological changes were observed in the heart, liver, spleen, lung and kidney (Fig. 7c). These results indicated that CR3022/B38 NVs have good biocompatibility.

## Conclusions

In conclusion, we designed a type of nanovesicle for the treatment of COVID-19. These nanovesicles were capable of neutralizing SARS-CoV-2 spike-pseudotyped viruses and targeted delivery of antiviral drugs. As CR3022/B38 NVs have a dual-target structure and do not contain a Fc domain, viral resistance to antibody-mediated neutralization and ADE effect may be avoided when neutralizing SARS-CoV-2. It is well known that many variants have emerged as a result of the ongoing SARS-CoV-2. In this case, monospecific nanovesicles may not be able to neutralize viruses well. However, bispecific nanovesicles are still able to neutralize the virus because the other scFv on its surface can bind to the unmutated epitope. In addition, the ADE effect caused by insufficient neutralization of nanovesicles was further reduced due to the enhanced binding ability of bispecific nanovesicles to S protein. Moreover, compared with the method of mixing two monospecific nanovesicles together, the co-expression of two scFvs on one novesicle is convenient for manufacturing and regulatory purposes. Furthermore, remdesivir loaded into CR3022/B38 NVs could be delivered to sites where SARS-CoV-2 has aggregated, and the drug plays a synergistic therapeutic role by inhibiting viral replication.

Importantly, this kind of nanovesicles holds great potential for the treatment of viral infections due to their modification possibilities. First of all, the drugs delivered by CR3022/B38 NVs are not limited to remdesivir. The nanovesicles could also be loaded with other antiviral drugs, such as free fatty acids [37], lopinavir, or ritonavir [38], allowing synergistic inhibition of SARS-CoV-2 with remdesivir. In addition, the scFv on nanovesicles targeting SARS-CoV-2 could be optimized through substitution and combination, or could even be replaced with anti-IL-6R scFv to reduce the production of inflammatory factors while neutralizing SARS-CoV-2 [39, 40]. However, more studies are needed to further transform the NV programming technique into clinical practice. In line with this, the neutralization and inhibitory ability of CR3022/B38 NVs loaded with remdesivir on SARS-CoV-2 should be tested under biosafety level 3 conditions. Second, the clearance ability of nanovesicles to eliminate SARS-CoV-2 needs to be tested in non-human primate (NHP) models or patients with COVID-19. Finally, to further improve the safety and uniformity of nanovesicles, researchers should pay more attention to the nanovesicles derived from different cell types and the unknown proteins present on the cell membrane [41].

## Methods and materials

### Cells and plasmids

LLC and HEK 293 T cells (ATCC) were cultured in Dulbecco’s Modified Eagle’s Medium (DMEM, Thermo Fisher Scientific) containing 10% FBS (Gibco, USA).

The nucleotide sequences encoding hACE2, S protein, and the three CARs were synthesized by BGI (Beijing, China). PCDH-ACE2-puro was obtained by cloning the ACE2 sequence into the pCDH-CMV-MCS-EF1-GFP-Puro vector (cat. no. CD511B-1; System Biosciences, Mountain View, CA, US). We obtained pCDH-S-puro and pcDNA3.1-S by cloning the S protein gene sequence into the pCDH-CMV-MCS-EF1-MEM-GFP-Puro vector modified in our laboratory and the pcDNA3.1 vectors. We obtained pCDH-CAR and pCDH-CAR-puro by cloning the three CAR gene sequences into pCDH-CMV-MCS-EF1-copGFP vector (System Biosciences) and pCDH-CMV-MCS-EF1-GFP-Puro lentiviral vector.

Plasmids were combined with pCDH-ACE2-puro, pCDH-S-puro, pCDH-CAR, and pCDH-CAR-puro with the helper plasmid psPAX2 (12,260; Addgene), and pMD2.G (12,259; Addgene) at a ratio of 4:3:1. The DNA mixture was mixed with polyethyleneimine (PEI) for 20 min and then co-transfected into 293 T cells to produce lentivirus LV-ACE2-puro, LV-S-puro, LV-CAR, and LV-CAR-puro. As previously reported [42], to obtain pseudotyped lentiviral particles with Spike protein, pcDNA3.1-S, pHIV-GFP-luc expression vector, pgagpol HIV vector, pHIV-Rev, and pHIV-TAT were co-transfected into 293 cells. After 48 h, the lentiviral virus was collected in the supernatant of the medium. The lentivirus was concentrated by polyethylene glycol 8000 (PEG 8000) precipitation and the titer was determined using a colloidal gold kit (Biodragon, Beijing, China).

Next, 293 T cells were transfected with LV-ACE2-puro, LV-S-puro, and LV-CAR-puro, and the cell line was screened with puromycin to produce 293 T-ACE2 cells, 293 T-S cells, and CAR-293 T cells.

### Preparation of CAR-T cells

The sequence of CR3022 (GenBank accession number of VH and VL: DQ168569, DQ168570) and B38 antibodies were synthesized by BGI (Beijing, China) [4], and the sequence of CR3022/B38 was obtained by combining CR3022 and B38 gene fragments using a linker. CD8 signal peptides were occupied at the front ends of CR3022-scFv, B38-scFv, and CR3022/B38-scFv to produce CR3022-CAR, B38-CAR, and CR3022/ B38-CAR, respectively, and the back ends were connected to MYC tags, CD8 hinges, transmembrane domains, a 4-1BB transactivation domain, and a CD3 zeta signaling domain [23]. CARs were cloned into the lentiviral backbone plasmid pCDH to produce pCDH-CR3022, pCDH-B38, and pCDH-CR3022/B38. Then pCDH-CARs, pspax2, and pMD2.G were co-transfected into 293 T cells at a ratio of 4:3:1, and lentiviruses in the supernatant were collected 48 h later.

T cells for CAR-T cell preparation were isolated from the peripheral blood of healthy volunteers. Peripheral blood mononuclear cells were collected by density gradient centrifugation in peripheral blood using Ficoll-Paque (GE Healthcare), and CD3^+^ T cells were obtained by negative selection using the RosetteSep Kit (Stem Cell Technologies). All samples were collected by the Review Committee of the Fifth Affiliated Hospital of Sun Yat-sen University following the approved protocol and written informed consent was obtained from each donor. T cells were then cultured in X-VIVO 15 (Lonza) medium containing 10% FBS and 100 U/mL recombinant human IL-2 (Peprotech). After T cells were stimulated with the T cell activator CD3/CD28 (Peprotech) for 48 h, concentrated lentivirus was added to the culture dish to infect T cells with a multiplicity of infection (MOI) of 10 [43]. CAR-T cells obtained after infection were cultured for 2 weeks to amplify CAR-T cells by 1,000 times, which were then used to prepare nanovesicles.

### Preparation of nanovesicles

As previously described [16], nanovesicles were prepared from CAR-T and 293 T-S cells. After washing three times with PBS, the cells were resuspended in a separation buffer solution containing 1 mM NaHCO_3_, 0.2 mM EDTA, and 1 mM PMSF, and the cell suspension was kept overnight at 4 °C. Next, the cells were ground 20 times with a Dounce homogenizer. Following disruption of the cells, the mixture was centrifuged at 800 × *g* for 5 min to remove large fragments, and the supernatant was collected. Next, the supernatant was centrifuged at 10,000 × *g* for 25 min, and the sediment was discarded. The supernatant was further centrifuged at 100,000 × *g* for 30 min, and the grayish-white membrane precipitate was collected. Finally, the obtained membrane was sequentially extruded 20 times through 800 and 200 nm polycarbonate membranes using an extruder.

### Flow cytometry

As previously reported [44], the expression of CARs in cells was measured using biotinylated protein L (GenScript, Piscataway, NJ, USA). Briefly, 1 μL of protein L (1 mg/mL) was added to 1 × 10^6^ cells and incubated at 4 °C for 30 min. The samples were washed three times with PBS containing 1% bovine serum albumin (BSA), then stained with PE-conjugated streptavidin (Biolegend, San Diego, CA, USA) and washed. To detect the expression of CARs, nanovesicles were coated with 4 μm aldehyde/sulfate latex beads (catalog no. A37304, Invitrogen) [45]; 10 μL of nanovesicles and 5 μL of latex beads were incubated at room temperature for 15 min. Then, 1 mL of PBS was added, and the mixture was rotated in a rotator for 2 h. Next, 110 μL glycine (100 mM) was added and incubated at room temperature for 30 min to block the remaining binding sites. The beads were then collected by centrifugation at 5000 rpm for 5 min and stained for flow cytometry. The flow cytometer used was a Cytoflex LX (Beckman Coulter, Atlanta, GA, USA). Cytexpert (Version 2.3) software was used to analyze the data obtained.

### TEM

The morphology and size of the nanovesicles were measured by TEM. Briefly, 10 μL of nanovesicles resuspended in PBS were dropped onto copper grids and negatively stained with 2% uranyl acetate aqueous solution for 2 min, and the excess solution was absorbed using filter paper. The samples were allowed to air dry, and electron micrographs were taken by TEM (JM-1400; JEOL, Tokyo, Japan) at 120 kV.

### NTA

The size distribution and particle concentration of nanovesicles were measured using a NanoSight NS300 system (Malvern Instruments Company, NanoSight, and Malvern, UK). Purified nanovesicles were mixed by vibration, double-diluted with PBS, and added to the NanoSight sample chamber for measurement. Data analysis was performed using NTA 3.0 analysis software (Malvern).

### Western blot analysis

Cells and extruded nanovesicles were treated with a lysis buffer (1% Triton X-100, 0.1% SDS, 0.1 M Tris HCl, pH 7) and protease inhibitor, and the total protein concentration was measured using a Micro BCA protein analysis kit (Pierce, Rockford, IL, USA). After resolution on a 10% SDS-PAGE gel, the samples were transferred to a PVDF membrane, blocked in 5% skim milk for 60 min, and washed twice with PBST. Then, the membrane was incubated overnight with an anti-MYC label antibody (Arigo Biolaboratories Corp.) at 4 °C. Following incubation, the HRP-conjugated secondary antibody (Cell Signaling Technology) was added to the membrane for 1 h at room temperature, and the membrane was developed by ECL chemiluminescence.

### Biological behaviors of nanovesicles in vitro

Free NVs, CR3022 NVs, B38 NVs, and CR3022/B38 NVs labeled with red fluorescent dye DIL (Abmole, USA) were separately co-incubated with 293 T-S cells to study the targeting of nanovesicles to 293 T-S cells. DIL labeling was performed as previously described. Briefly, 10 μg of nanovesicles were stained with 1 μL DIL at 37 °C for 30 min, before the mixture was centrifuged at 120,000 × g for 90 min to remove the free dye. The labeled nanovesicles were washed twice and then resuspended in 200 uL of PBS. Both 1 μg DIL labeled nanovesicles and 293 T-S cells were incubated in confocal culture dishes for 4 h, and the cells were washed twice with PBS and fixed for 30 min with 4% paraformaldehyde. The nucleus of 293 T-S cells was stained with the purple fluorescent dye DAPI (Abmole, USA). The binding of nanovesicles and 293 T-S cells after co-incubation was observed using a Zeiss LSM 880 confocal microscope.

### Neutralization experiment

Pseudotyped lentiviral particles expressing spike protein and 293 T-ACE2 cells were obtained as described above.

The infectious ability of the spike-pseudotyped virus was determined by infecting 293 T and 293 T-ACE2 cells. Briefly, 293 T-ACE2 cells (2 × 10^4^) were seeded into poly (L-lysine)-coated 96-well plates and cultured overnight at 37 °C in an incubator. After approximately 12 h, 1 × 10^5^ spike-pseudotyped virus and serial twofold dilutions of the three types of nanovesicles were incubated at 4 °C for 10 min and then added to 96-well plates. ACE2-NVs and CR3022/B38 NVs were mixed at a ratio of 1:1 for the neutralization experiment. After 36 h, luciferase expression in 293 T-ACE2 cells was measured using the bright-GlO luciferase assay system based on the manufacturer’s instructions (E2610, Promega, Madison, WI, USA), and the neutralization ability of nanovesicles was assessed by calculating the half-maximal inhibitory concentration (IC50). Luminescence was detected using an EnVision Multilabel Plate Reader (Perkin Elmer).

### Loading of remdesivir into nanovesicles

Remdesivir was loaded into nanovesicles using electroporators (Scientz-2C, Ningbo SCIENTZ Biotech Co, Ltd., Ningbo, China). Briefly, 20 μg of nanovesicles (1 μg/μL) and 10 μg of remdesivir were mixed with 200 μL PBS buffer containing 25 mM trehalose at 4 °C [46]. Electroporation was performed under 125 μF, 350 v, and 400 Ω. After electroporation, the mixture was incubated at 37 °C for 30 min to fully recover the nanovesicle membrane. Next, the nanovesicles carrying remdesivir were dissolved in PBS and centrifuged for 90 min at 120,000 × *g* to remove free remdesivir. Finally, the specific absorption peak of remdesivir at 247 nm was measured by spectrophotometry, and the mass of remdesivir encapsulated in nanovesicles was calculated. The amount of loaded remdesivir was assessed using the following equation: W_Remdesivir_/W_NVs_ × 100%. The nanovesicles loaded with remdesivir were placed in the dialysis membrane with a molecular weight cut-off of 25 k (Solarbio, Beijing, China). Remdesivir released from the dialysis bag was collected and tested at different time points to determine the efficiency with which the drug was released.

### Calcein-AM/PI staining

For calcein-AM/PI staining, 293 T or 293 T-S cells were seeded into 12-well plates at a density of 2 × 10^5^ cells per well and incubated for 16 h. Then, 12 μL of PBS, 12 μg of free NVs, 10 μL of PBS containing 2 μg of remdesivir, 10 μg of free NVs containing 2 μg of remdesivir, and 10 μg CR3022/B38 NVs containing 2 μg of remdesivir were added to the plates. After 10 h, the old medium was discarded, and the cells were washed twice with PBS. Based on the reagent manufacturer’s instructions, 200 μL of Calcein-AM/PI was added to the cells of different treatment groups, incubated at 37 °C for 20 min, and observed through an inverted fluorescence microscope. Green fluorescence represents living cells, and red fluorescence represents dead cells.

### Animal experiments

All animal experiments were approved by the Fifth Affiliated Hospital of Sun Yat-sen University and Animal Care and Use Occasion. Six-week-old female C57BL/6 J mice were purchased from the Guangdong Provincial Medical Animal Center. The constructed LLC-ACE2 or LLC-S cells (2 × 10^6^ cells suspended in 200 μL of PBS) were subcutaneously injected into the lower left groin of the mice, and the animal model required for the experiment was ready approximately 2 weeks later.

Free or CR3022/B38 NVs were stained with 5 µmol/L of DiR (Abmole, USA) at 37 °C for 30 min. Free DiR was removed by centrifugation at 120,000 × g for 90 min, and the nanovesicles labeled with DiR dye were collected. 200 μg of free or CR3022/B38 NVs labeled with DiR were injected into LLC-S tumor-bearing mice through the tail vein. The mice were dissected 12 h post-injection, and the distribution of free NVs or CR3022/B38 NV in LLC-S tumor tissue and mouse organs was measured by IVIS (Xenogen).

To test the neutralization of CR3022/B38 NVs in vivo, 100 μL of 2.5 × 10^7^ TU/mL Spike-pseudotyped virus was injected into LLC-ACE2 bearing mice through the tail vein. Then, the administration of 200 μg of free or CR3022/B38 NVs was performed at 12 h, 2 h, and 0 h before or 6 h post-challenge with pseudoviruses. Seventy-two hours after pseudovirus injection, the mice were anesthetized with isoflurane, and each mouse was intraperitoneally injected with 3 mg of luciferin (Abmole, US). The bioluminescence intensity of LLC-ACE2 tumor sites was measured 15 min later using IVIS (Xenogen) to evaluate the neutralization ability of free or CR3022/B38 NVs against the spike-pseudotyped virus.

### Safety assessment of nanovesicles

The in vitro cytotoxicity of CR3022/B38 NVs was assessed by a CCK-8 assay, as previously reported [47]. Human normal liver (L02) cells and human proximal tubular epithelial (HK-2) cells were seeded into a 96-well plate at a density of 1 × 10^4^ cells per well and incubated at 37 °C, 5% CO_2_ for 24 h. CR3022/B38 NVs were diluted with DMEM medium to obtain concentrations in the range of 0.05, 0.1, 0.5, 1, 1.5 and 2 mg/mL. The cells were co-cultured with diluted nanovesicles for another 48 h and the cell viability was measured by CCK-8 (Beyotime, Shanghai, China) assays.

To evaluate the biological toxicity of NVs after multiple administration, C57 mice were injected with 200 μg CR3022/B38 NVs or 200 μL PBS via the caudal vein on days 0, 3, 6, 9, and 12. The body weight of the mice was monitored continuously for 15 days from day 0 of injection, and the mice were killed at day 15 to observe the effect of nanovesicles on the morphology of mice tissue. Hematoxylin and eosin (H&E) staining was performed to observe the tissue morphology. In brief, the major organs, such as the heart, liver, spleen, lung, and kidney were collected and fixed with formalin, 10% neutral buffered formalin, paraffin-embedded, and stained by H&E according to a routine protocol. The sections were examined under a light microscope to assess the pathological changes of major organs.

### Statistical analysis

All data were analyzed using GraphPad Prism 5.0. All data are expressed as the mean ± s.d. All comparisons between two groups were analyzed by Student’s t-test. *P-values* < 0.05 were considered statistically significant.

## Supplementary Information

**Additional file 1: Fig. S1. a** GFP fluorescence localization on the 293T cell membrane. **b** Detection of S protein expression in 293T cells by Western blot analysis. **c** Detection of ACE2 protein expression in 293T cells by Western blot analysis. **Fig. S2. **The IC50 of CR3022-293T NVs, B38-293T NVs and CR3022/B38-293T NVs was detected by measuring the luciferase expression levels in 293T-ACE2 cells. **Fig. S3. **The zeta-potential of nanovesicles or nanovesicles loaded with remdesivir were measured using a Malvern Zetasizer Nano ZSP (*n*= 3, error bar, mean±s.d). **Fig. S4. **Storage stability of nanovesicles or nanovesicles loaded with remdesivir at – 80 °C. The average size of nanovesicles loaded with remdesivir did not change within 28 days at – 80 °C (*n*= 3, error bar, mean±s.d). **Fig. S5. **The stability of nanovesicles in PBS buffer and PBS buffer with 20% of fetal bovine serum (FBS) were measured using a Malvern Zetasizer Nano ZSP (*n*=3, error bar, mean±s.d). **Fig. S6. **Quantitative analysis of cell survival rate by Calcein AM/PI staining (*n*=3, error bar, mean±s.d). ***P<0.001. **Fig. S7. **Neutralization ability of CR3022/B38 NVs in vivo. 12 and 0 hours before Spike-pseudotyped viruses injection or 6 hours after Spike-pseudotyped viruses administration, the LLC-ACE2 tumor-bearing mice were injected with free and CR3022/B38 NVs through the tail vein, respectively. 72 hours after Spike-pseudotyped viruses injection, luciferase intensity was quantified by IVIS imaging.

## Data Availability

The datasets used and/or analyzed during the current study are available from the corresponding author on reasonable request.

## Abbreviations

COVID-19: Coronavirus disease 2019
SARS-CoV-2: Severe acute respiratory syndrome coronavirus 2
ACE2: Angiotensin-converting enzyme 2
CAR-T: Chimeric antigen receptor T cell
CRS: Cytokine release syndrome
IC50: Half maximal inhibitory concentration
ScFv: Single-chain fragment variables
293 T-S: 293 T cells expressing S protein
293 T-ACE2 cells: 293 T cells with high levels of expression of ACE2
S-NVs: Nanovesicles expressing spike protein
MOI: Multiplicity of infection
TEM: Transmission electron microscopy
NTA: Nanoparticle tracking analysis
