# Supplementary material for: Nanovesicles derived from bispecific CAR-T cells targeting the spike protein of SARS-CoV-2 for treating COVID-19
Source: J Nanobiotechnology. 2021 Nov 25;19:391. doi: 10.1186/s12951-021-01148-0 (PMC8614633; doi:10.1186/s12951-021-01148-0)
Supplement: Supplementary file 1 — Additional file 1: Fig. S1. a GFP fluorescence localization on the 293T cell membrane. b Detection of S protein expression in 293T cells by Western blot analysis. c Detection of ACE2 protein expression in 293T cells by Western blot analysis. Fig. S2. The IC50 of CR3022-293T NVs, B38-293T NVs and CR3022/B38-293T NVs was detected by measuring the luciferase expression levels in 293T-ACE2 cells. Fig. S3. The zeta-potential of nanovesicles or nanovesicles loaded with remdesivir were measured using a Malvern Zetasizer Nano ZSP (n= 3, error bar, mean±s.d). Fig. S4. Storage stability of nanovesicles or nanovesicles loaded with remdesivir at – 80 °C. The average size of nanovesicles loaded with remdesivir did not change within 28 days at – 80 °C (n= 3, error bar, mean±s.d). Fig. S5. The stability of nanovesicles in PBS buffer and PBS buffer with 20% of fetal bovine serum (FBS) were measured using a Malvern Zetasizer Nano ZSP (n=3, error bar, mean±s.d). Fig. S6. Quantitative analysis of cell survival rate by Calcein AM/PI staining (n=3, error bar, mean±s.d). ***P<0.001. Fig. S7. Neutralization ability of CR3022/B38 NVs in vivo. 12 and 0 hours before Spike-pseudotyped viruses injection or 6 hours after Spike-pseudotyped viruses administration, the LLC-ACE2 tumor-bearing mice were injected with free and CR3022/B38 NVs through the tail vein, respectively. 72 hours after Spike-pseudotyped viruses injection, luciferase intensity was quantified by IVIS imaging. [file 12951_2021_1148_MOESM1_ESM.docx]

**Additional file 1 for**

**Nanovesicles Derived from Bispecific CAR-T Cells Targeting the Spike Protein of SARS-CoV-2 for Treating COVID-19**

Tianchuan Zhu ^a,b,c,d,†^, Yuchen Xiao ^a,†^, Xiaojun Meng ^a,b,†^, Lantian Tang ^a^, Bin Li ^a^, Zhaoyan Zhao ^a^, Qingqin Tan ^a^, Lei Liu ^d,^*, Hong Shan ^b,c,^*, and Xi Huang ^a,b,c,d,^*

^a^ Center for Infection and Immunity, The Fifth Affiliated Hospital of Sun Yat-sen University, Zhuhai 519000, Guangdong, China

^b^ Guangdong Provincial Key Laboratory of Biomedical Imaging, The Fifth Affiliated Hospital of Sun Yat-sen University, Zhuhai 519000, Guangdong, China

^c^ Southern Marine Science and Engineering Guangdong Laboratory, Zhuhai 519000, Guangdong, China

^d^ Shenzhen Key Laboratory of Pathogen and Immunity, National Clinical Research Center for Infectious Disease, Shenzhen Third People's Hospital, Shenzhen 518112, Guangdong, China

* Corresponding author: [huangxi6@mail.sysu.edu.cn](mailto:huangxi6@mail.sysu.edu.cn) (X. Huang)

shanhong@mail.sysu.edu.cn (H. Shan), liulei3322@aliyun.com (L. Liu)

^†^ These authors contributed equally to this work.

**Additional file 1 figures**

**
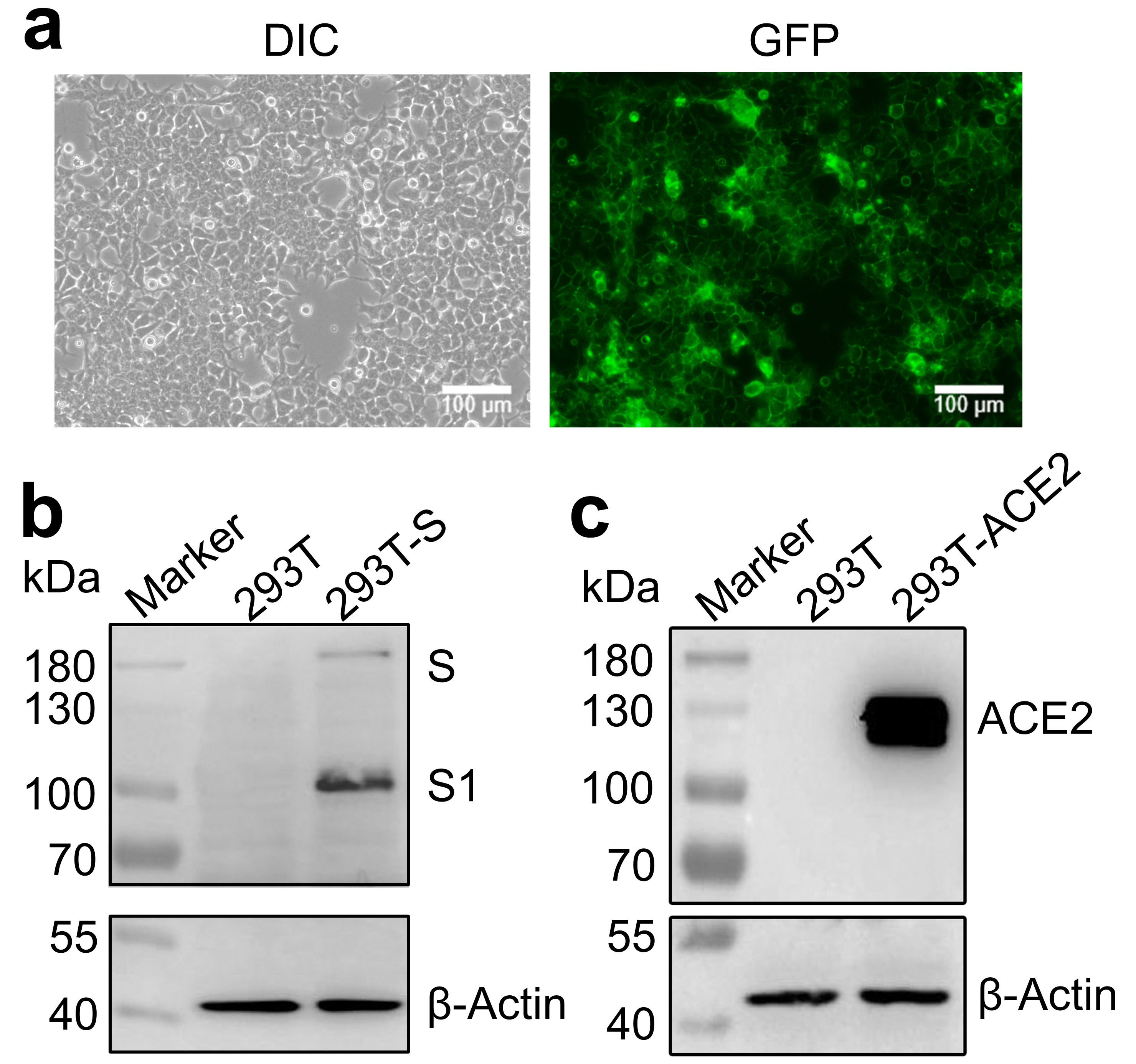
**

**Fig. S1.** a) GFP fluorescence localization on the 293T cell membrane. b) Detection of S protein expression in 293T cells by Western blot analysis. c) Detection of ACE2 protein expression in 293T cells by Western blot analysis.

**
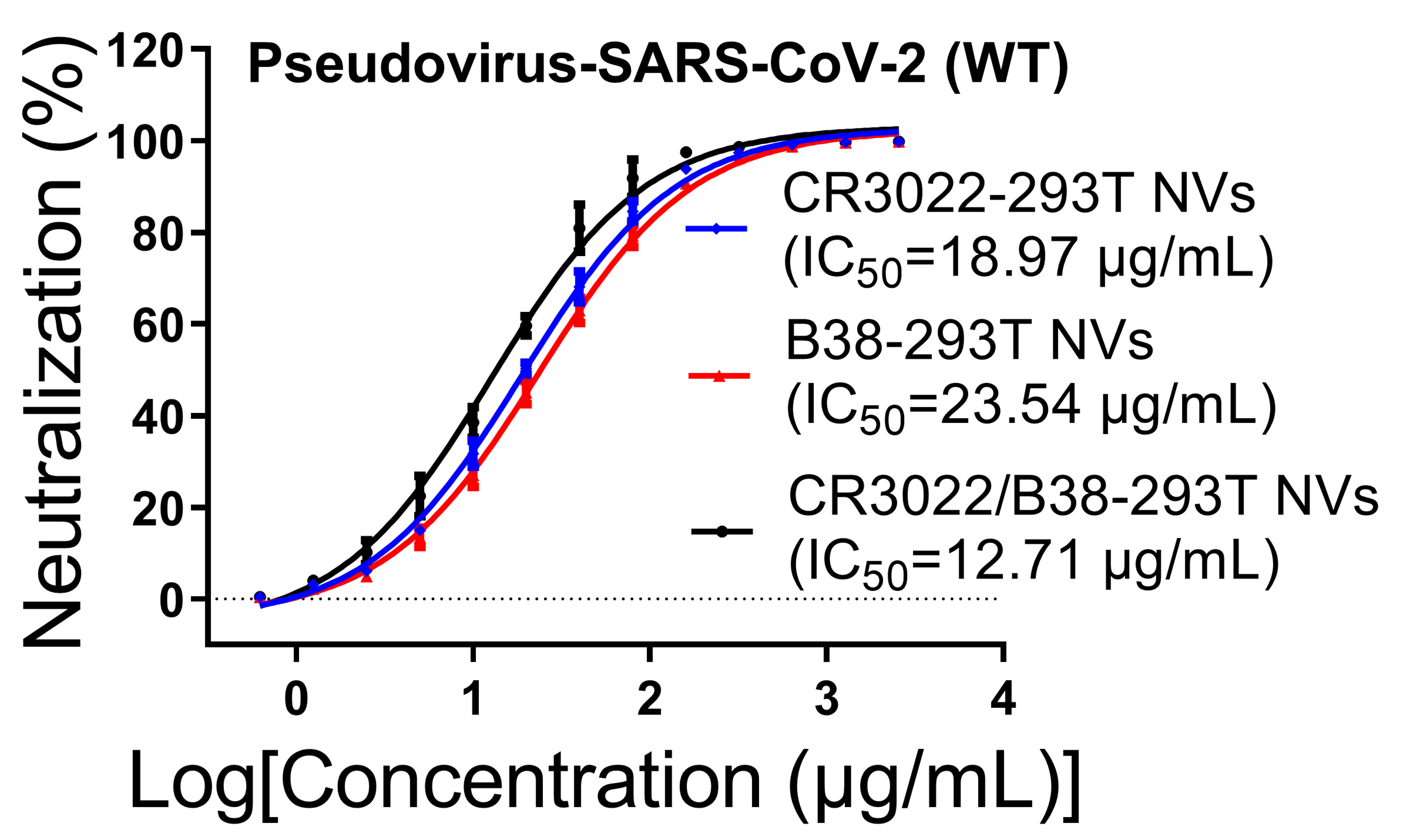
**

**Fig. S2.** The IC50 of CR3022-293T NVs, B38-293T NVs and CR3022/B38-293T NVs was detected by measuring the luciferase expression levels in 293T-ACE2 cells.


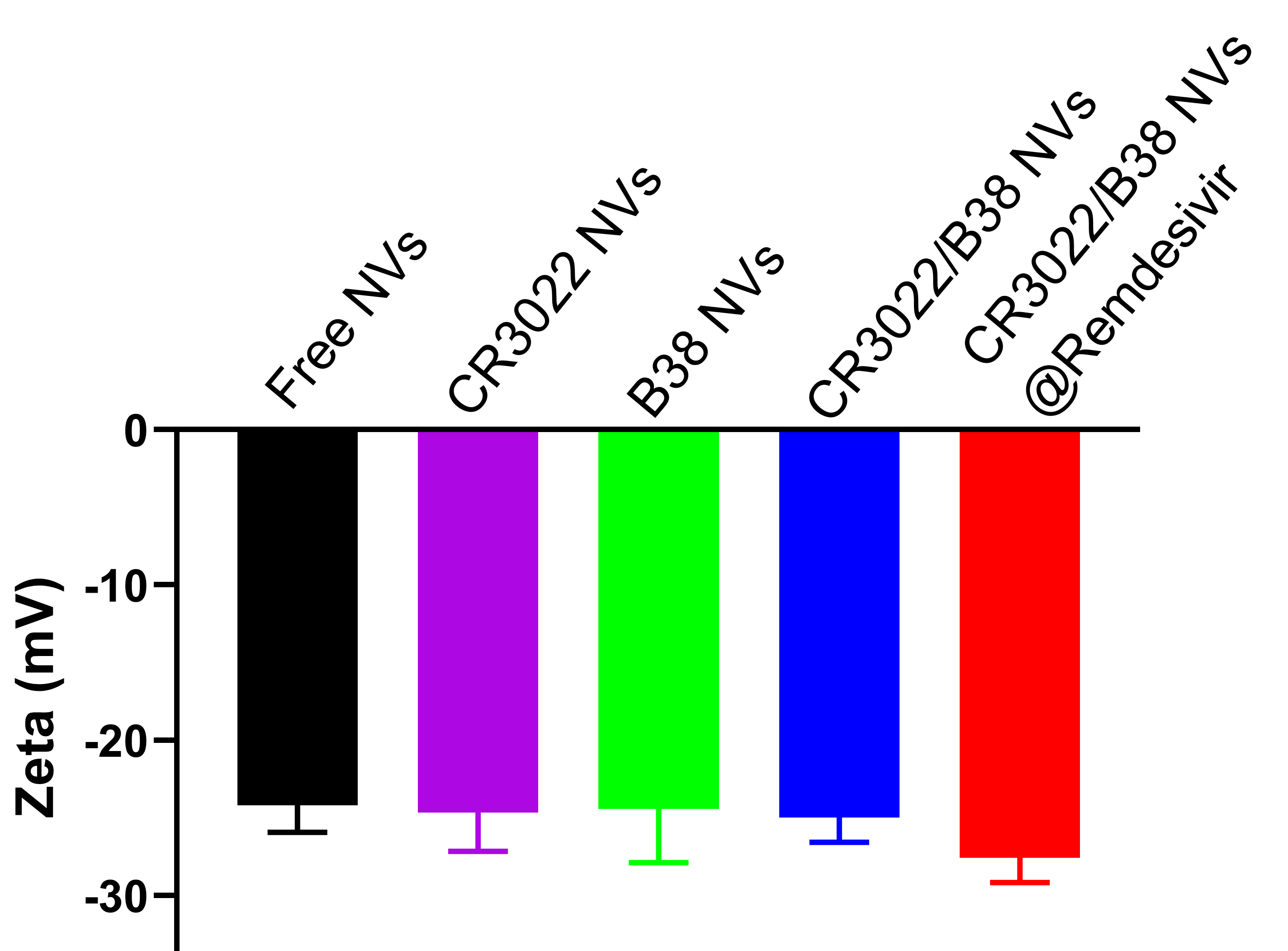


**Fig. S3.** The zeta-potential of nanovesicles or nanovesicles loaded with remdesivir were measured using a Malvern Zetasizer Nano ZSP (*n* = 3, error bar, mean ± s.d).


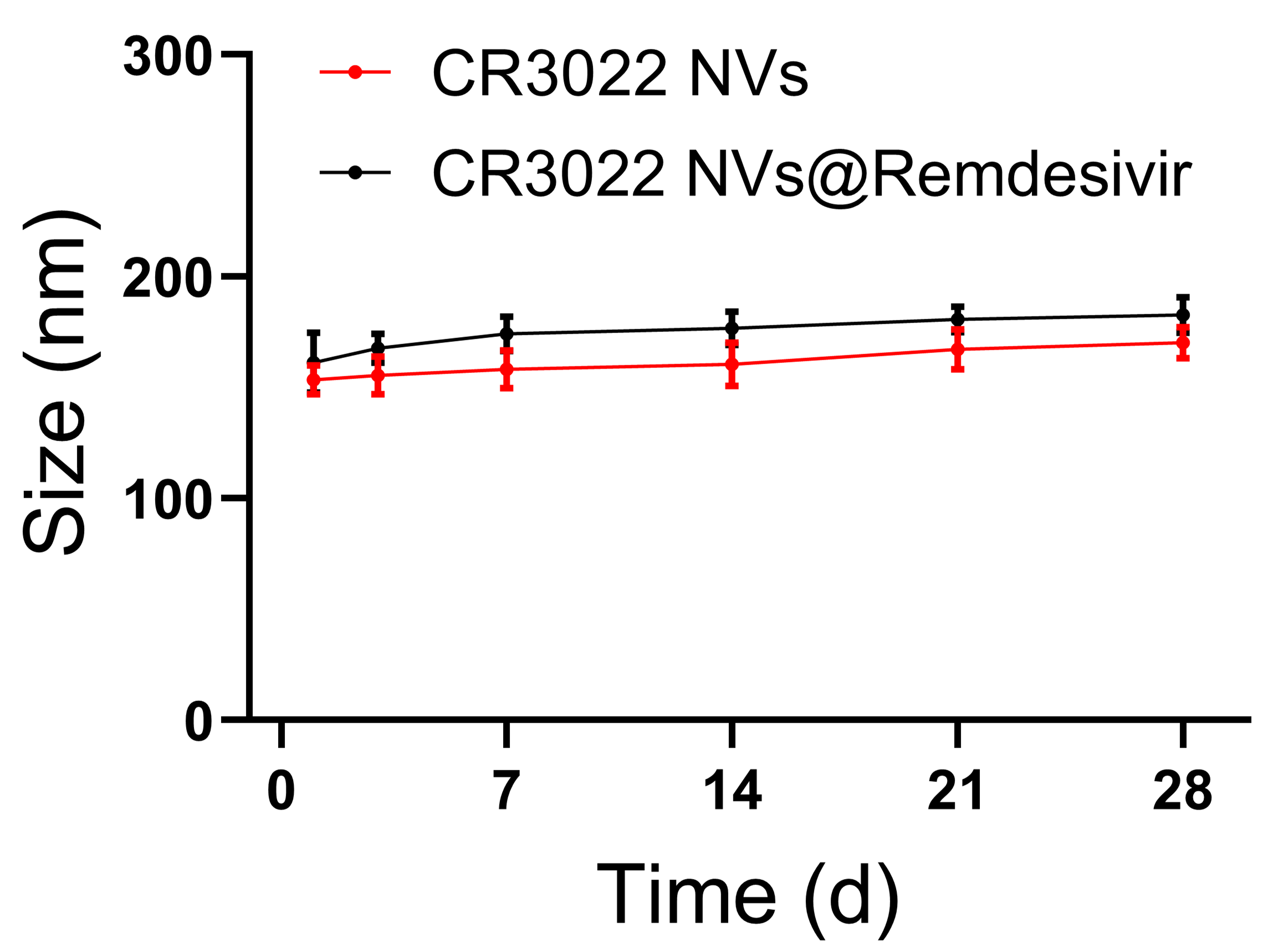


**Fig. S4.** Storage stability of nanovesicles or nanovesicles loaded with remdesivir at -80°C. The average size of nanovesicles loaded with remdesivir did not change within 28 days at -80°C (*n* = 3, error bar, mean ± s.d).


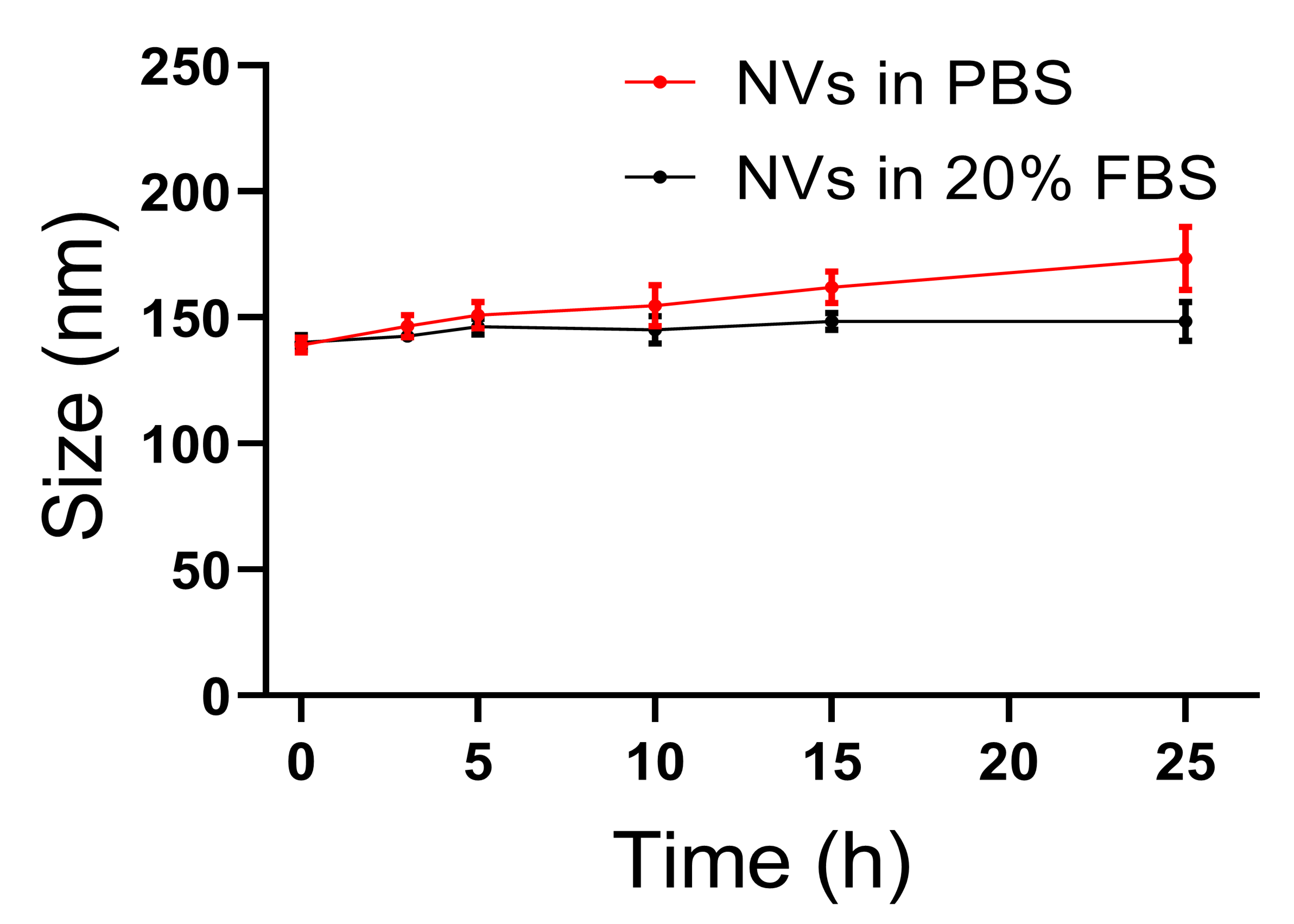


**Fig. S5.** The stability of nanovesicles in PBS buffer and PBS buffer with 20% of fetal bovine serum (FBS) were measured using a Malvern Zetasizer Nano ZSP (*n* = 3, error bar, mean ± s.d).


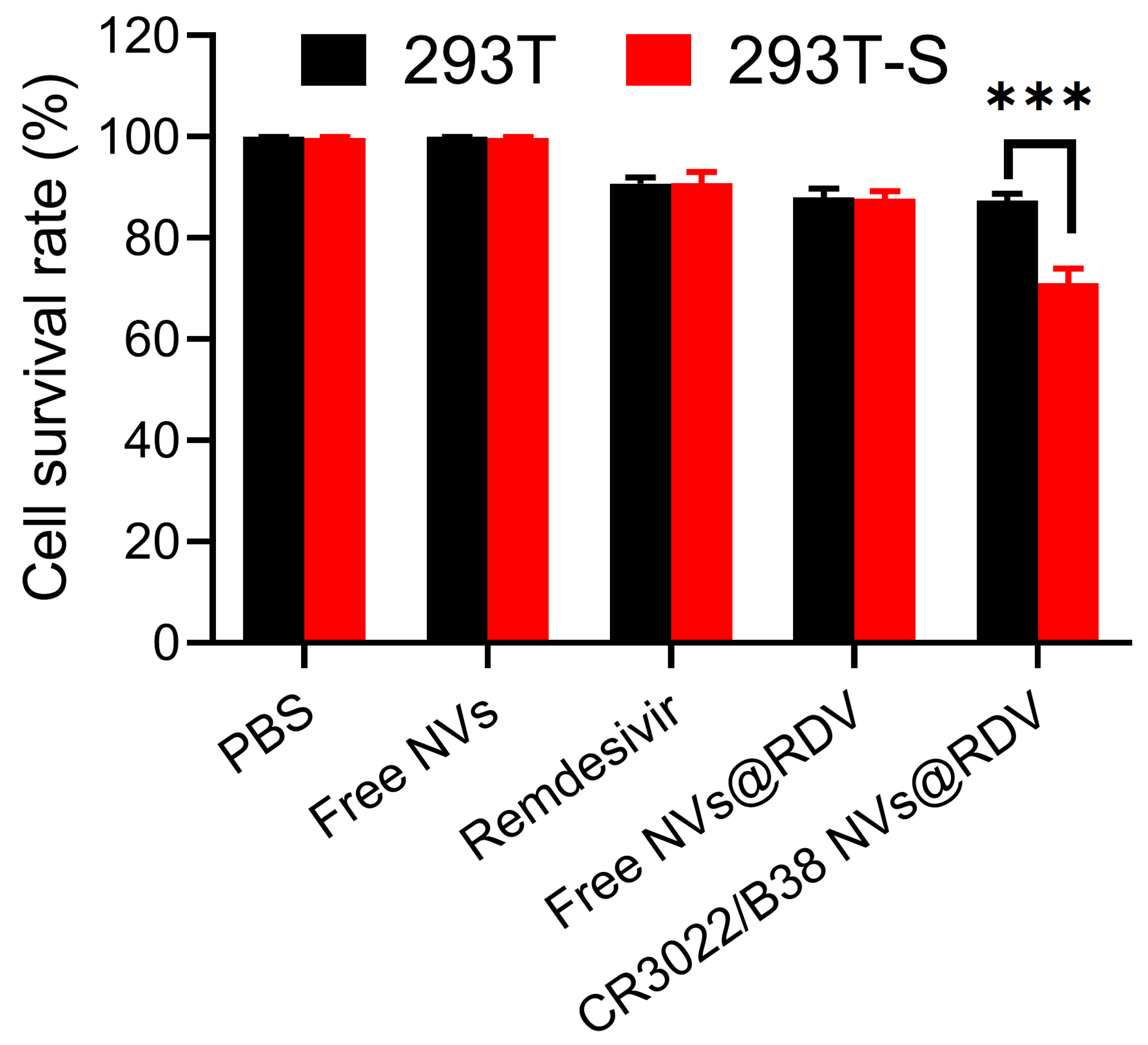


**Fig. S6.** Quantitative analysis of cell survival rate by Calcein AM/PI staining (*n* = 3, error bar, mean ± s.d). ****P*<0.001.


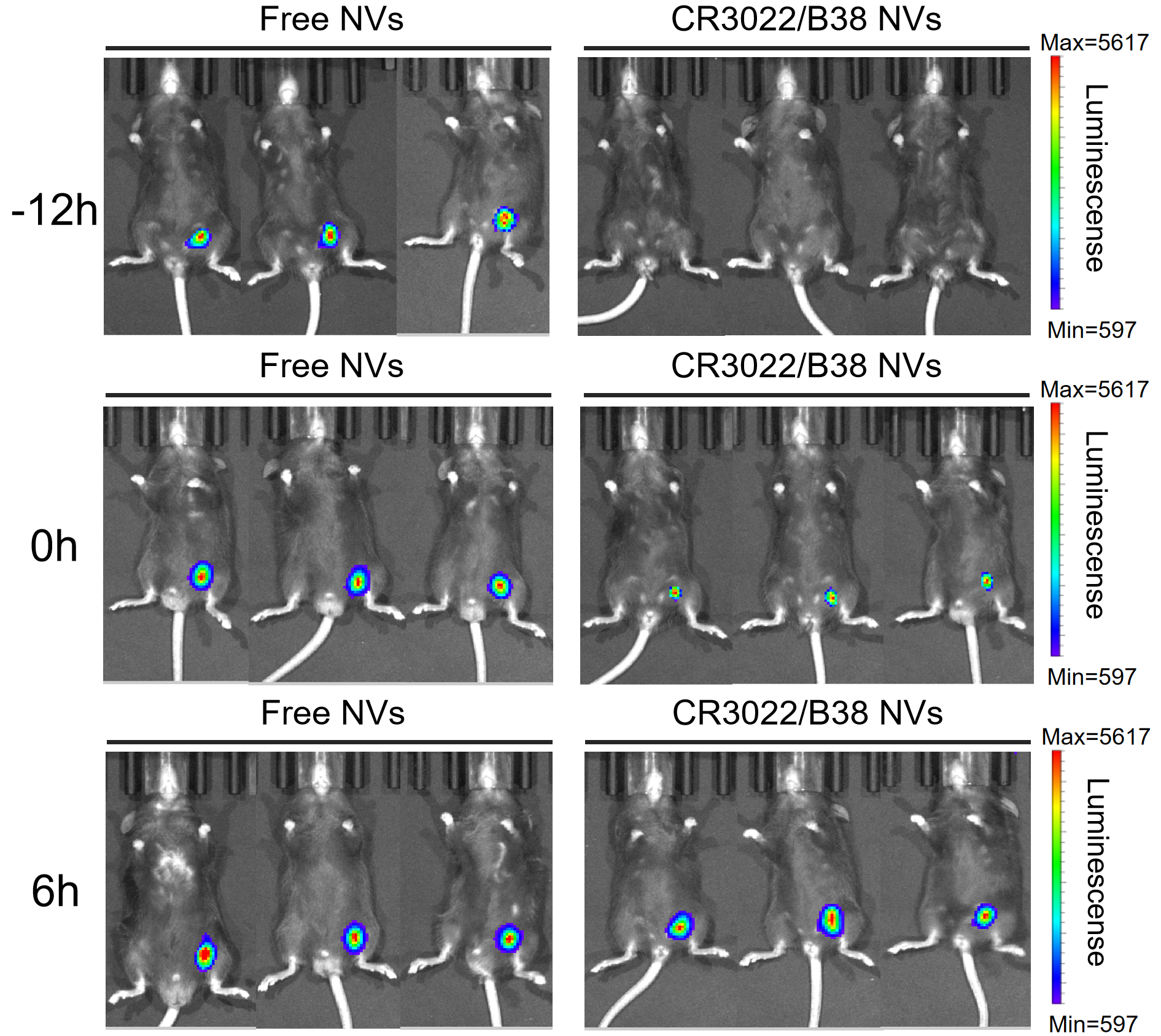


**Fig. S7. Neutralization ability of CR3022/B38 NVs *in vivo*.** 12 hours and 0 hours before Spike-pseudotyped viruses injection or 6 hours after Spike-pseudotyped viruses administration, the LLC-ACE2 tumor-bearing mice were injected with free and CR3022/B38 NVs through the tail vein, respectively. 72 hours after Spike-pseudotyped viruses injection, luciferase intensity was quantified by IVIS imaging.
